# Bioinformatic analysis of gene expression data reveals Src family protein tyrosine kinases as key players in androgenetic alopecia

**DOI:** 10.3389/fmed.2023.1108358

**Published:** 2023-06-09

**Authors:** Adaikalasamy Premanand, Baskaran Reena Rajkumari

**Affiliations:** Department of Integrative Biology, School of Bio Sciences and Technology, Vellore Institute of Technology, Vellore, Tamil Nadu, India

**Keywords:** androgenetic alopecia, differential gene expression analysis, reactome functional interaction network, STRING protein-protein interaction network, gene ontology, motif analysis, Wnt/β-catenin signaling, Src family protein tyrosine kinases

## Abstract

**Introduction:**

Androgenetic alopecia (AGA) is a common progressive scalp hair loss disorder that leads to baldness. This study aimed to identify core genes and pathways involved in premature AGA through an *in-silico* approach.

**Methods:**

Gene expression data (GSE90594) from vertex scalps of men with premature AGA and men without pattern hair loss was downloaded from the Gene Expression Omnibus database. Differentially expressed genes (DEGs) between the bald and haired samples were identified using the *limma* package in R. Gene ontology and Reactome pathway enrichment analyses were conducted separately for the up-regulated and down-regulated genes. The DEGs were annotated with the AGA risk loci, and motif analysis in the promoters of the DEGs was also carried out. STRING Protein-protein interaction (PPI) and Reactome Functional Interaction (FI) networks were constructed using the DEGs, and the networks were analyzed to identify hub genes that play could play crucial roles in AGA pathogenesis.

**Results and discussion:**

The *in-silico* study revealed that genes involved in the structural makeup of the skin epidermis, hair follicle development, and hair cycle are down-regulated, while genes associated with the innate and adaptive immune systems, cytokine signaling, and interferon signaling pathways are up-regulated in the balding scalps of AGA. The PPI and FI network analyses identified 25 hub genes namely CTNNB1, EGF, GNAI3, NRAS, BTK, ESR1, HCK, ITGB7, LCK, LCP2, LYN, PDGFRB, PIK3CD, PTPN6, RAC2, SPI1, STAT3, STAT5A, VAV1, PSMB8, HLA-A, HLA-F, HLA-E, IRF4, and ITGAM that play crucial roles in AGA pathogenesis. The study also implicates that Src family tyrosine kinase genes such as LCK, and LYN in the up-regulation of the inflammatory process in the balding scalps of AGA highlighting their potential as therapeutic targets for future investigations.

## Introduction

Androgenetic alopecia (AGA) is a complex genetic disorder characterized by a progressive loss of scalp hair leading to baldness. It is more prevalent in men than women, and the hair loss pattern differs between the sexes (1). In men, AGA, also known as male pattern hair loss, is defined by a distinct M-shaped pattern hair loss that begins with a bi-temporal recession of the frontal hairline, followed by hair thinning at the frontal and vertex scalp region, which eventually converges resulting in complete baldness in the frontal and vertex scalp region (1, 2). Hair loss, particularly adolescent AGA, causes serious psychosocial ramifications in men affecting their self-esteem and quality of life (3).

Hair loss in AGA is attributed to the gradual transformation of thick pigmented large terminal hairs into non-pigmented small fine vellus hair through hair follicle miniaturization process driven by the androgen 5α-dihydrotestosterone (5α-DHT) (1). However, the mechanism of hair follicle miniaturization is poorly understood and the inadequate understanding of the pathobiology of AGA impedes the search for a permanent cure to hair loss (4). Molecular genetic studies have identified 12 genomic regions of interest and genes such as AR, EDA2R, PAX1, FOXA2, HDAC9, TARDBP, HDAC4, AUTS2, IMP5, SETBP1, SUCNR, MBBL1, EBF1, WNT10A, SSPN, and ITPR2 associated with AGA (2). However, these identified genes explain only a limited proportion of the pathogenesis and genetic variance of AGA since most of the identified genetic variants reside in the non-coding region of the genome for which no clear functional effect has been established yet (2). Hence, the identification of additional genetic loci for AGA is warranted to understand the pathobiology and to aid drug discovery.

Recently, Michel et al. (5) performed a microarray gene expression analysis between hairless or bald vertex scalp from young men with premature AGA and haired scalp from control men to identify dysregulated genes in AGA. The identification of differentially expressed genes (DEGs) was carried out by analysis of variance test and Tukey’s *post-hoc* tests. After Benjamini-Hochberg correction they, found 184 down-regulated and 149 up-regulated genes in the AGA group compared with the healthy group. In this study, we utilized the same data of Michel et al. (5) to identify DEGs in the AGA pathology employing a different method and threshold criteria. We constructed biological networks, such as the STRING protein-protein interaction (PPI) and Reactome Functional Interactome (FI) networks, using the DEGs obtained. We then focused on the hub nodes in both the PPI and FI networks and identified the hub genes that were common to both networks as worthy of further investigation into the signaling pathways involved in AGA development.

## Materials and methods

### Microarray data

The raw dataset of the gene expression profile GSE90594 generated by Michel et al. (5) was downloaded from the GEO database (6). The data was obtained from scalp biopsies taken from the vertex region of 14 young males with premature alopecia (age 29.4 ± 3.4 years, stage V–VII as per Hamilton-Norwood classification) and 14 healthy volunteers with less than 2% white hairs (age 26.1 ± 3.6 years, Stage I or II according to Hamilton- Norwood classification). Both the alopecia and healthy group did not have any other skin involvement, autoimmune disorders, and systemic diseases (5).

### Data preprocessing and differential gene expression analysis

*limma* v3.50.3 (Linear Models for Microarray Data) package, a R/Bioconductor software package, which provides an integrated solution for analyzing gene expression data from microarray technologies was utilized for data analysis (7). The Data pre-processing included background correction using normexp method and quantile normalization. Boxplot and cluster analyses were performed to identify and remove outliers in the samples. Then the control probes and the unexpressed probes are filtered out while the probes that are expressed above background are retained for further analysis. In addition, for multiple probes corresponding to the same genes in the arrays their average expression value was computed by *avereps* function in *limma*. Then the DEGs for the alopecia samples compared to the healthy samples were mined using the single-channel design matrix provided in the *limma* package. Benjamini and Hochberg’s method was utilized to compute the adjusted *p*-values (False Discovery Rate, FDR) (8). The probes with adjusted *p*-value (FDR) < 0.05 were selected as differentially expressed.

### Gene ontology and pathway enrichment analysis

ToppGene Suite^1^ (updated: Mar 2021) was employed to perform gene ontology (GO) functional and pathway analysis to identify items in gene lists that may have relevance to the biological question being investigated (9, 10). The ToppFun function in the ToppGene Suite was utilized to carry out GO (biological process and molecular function), gene family (source: genenames.org), and pathway enrichment (source: Biosystems-Reactome) analyses for the DEGs. All genes that are detected in the microarray analysis were used as background gene set in the ToppGene Suite for these analyses. The probability density function which is the default method for *p*-value and FDR calculation was selected. Gene count >2 and FDR B&H *q*-value < 0.05 were chosen as the cut-off criteria for the analyses.

### Annotation of differentially expressed genes with AGA risk loci

Windows of 50 kb, 100 kb and 500 kb flanking the 107 lead SNPs associated from 8 genome wide association studies [Study Accession IDs: GCST000250 (11), GCST000251 (12), GCST001548 (13), GCST001297 (14), GCST005116 (15), GCST90043616 (16), GCST003983 (17), and GCST90043619 (16)] for the trait androgenetic alopecia indexed in the NHGRI-EBI GWAS catalog were prepared by calculating coordinates of 50 kb, 100 kb and 500 kb distance on either sides from the SNP position. Gene coordinates of DEGs transcript(s) were annotated using RefSeq Identifiers (Hg38). The flanking coordinates of SNPs were overlapped with the coordinates of DEGs utilizing the intersect function in Bedtools v2.30.0 (18). An overlap is only considered when there is a minimum of 1 bp overlap between the coordinates of DEG transcripts and the flanking coordinates of the lead SNPs (19).

### Motif analysis in the promoter regions of differentially expressed genes

The promoter regions of up and down-regulated DEGs were separately subjected to motif analysis utilizing the gene-based analysis method in HOMER v4.11 software^2^ (20). 2,000 bp upstream and 200 bp downstream relative to the transcriptional start site of the genes were considered as promoter regions (19) and the promoter sets for the DEGs were constructed based on RefSeq genes (Hg38). Motifs of length up to 12 bases were probed with Benjamini-Hochberg-corrected *p*-value ≤ 0.05 as cut-off value.

### STRING protein-protein interaction network

The protein-protein interaction (PPI) interaction network for the DEGs were computed through the STRING database. The online web resource STRING v11.5^3^ is a biological database that includes direct (physical) and indirect (functional) protein-protein association data which are both specific and biologically meaningful (21). The PPI interaction network for the DEGs were computed through the stringApp plugin v1.7.1 in Cytoscape v3.9.1 (22). An interaction score of 0.900 (highest confidence) was used as the cut off criterion for constructing the PPI network.

### Reactome functional interaction network

Reactome functional interaction (FI) network was constructed for the DEGs utilizing the Cytoscape application ReactomeFIViz v8.0.4 which probe for disease-related pathways and network patterns using the Reactome functional interaction (FI) network (23, 24) created based on the well-known biological pathway database Reactome^4^ (25, 26). Reactome FI network 2021 version was used to construct the FI network for the DEGs. Gene ontology biological process and pathway enrichment analysis for the nodes (genes) mapped in the network was carried out through the inbuilt Reactome FI network analysis tool.

### Network analysis and hub gene identification

The topological properties of the PPI and FI network were analyzed through the Cytoscape pre-installed network analyzer v4.4.8 tool (27). Cytohubba v0.1 plugin was used to identify hub proteins in the PPI and FI network and rank them based on topological algorithms and centralities such as Maximal Clique Centrality (MCC), Maximum Neighborhood component (MNC), Density of Maximum Neighborhood Component (DMNC), Degree, Closeness, and betweenness (28). The clusters in the networks were determined using the MCODE plugin with specific parameters including a degree cut-off of 2, fluff node density cut-off of 0.1, node score cut-off of 0.2, K-core of 2, and max depth of 100 to determine the highly interconnected nodes (29).

## Results

### Data processing and screening of differentially expressed genes

The GSE90594 dataset contained 28 samples of which 14 samples are from men with premature AGA and 14 samples from healthy men without hair loss. Cluster analysis of the samples after background correction and normalization of the arrays revealed 9 samples (5 alopecia and 4 healthy samples) as outliers (Supplementary I-1, 2). The outlier samples were removed, and differential gene expression analysis was carried out between 9 alopecia and 10 healthy samples. The probes were annotated with Entrez Gene ID, Gene Symbol, and Gene names using the clusterProfiler 4.0 v4.4.3 package in R by querying the Reference Seq ID (30). From this list, the probes that have a valid Entrez Gene ID are selected for further analysis. Subsequently probes with similar Probe IDs (Probe Names) are averaged using avereps function in *limma* and the probes with different probe ID for same genes are kept as such.

**FIGURE 1:**
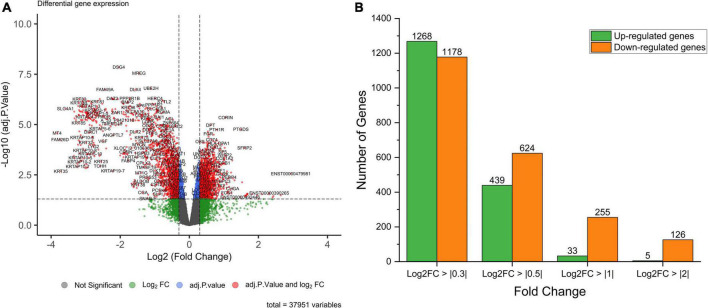
**(A)** Volcano plot of the differentially expressed genes in AGA with log_2_FC > | 0.3| and adj. *p*-value < 0.05 as cut off value. **(B)** Bar chart depicting the number of differentially expressed genes for the log_2_FC values.

**FIGURE 2:**
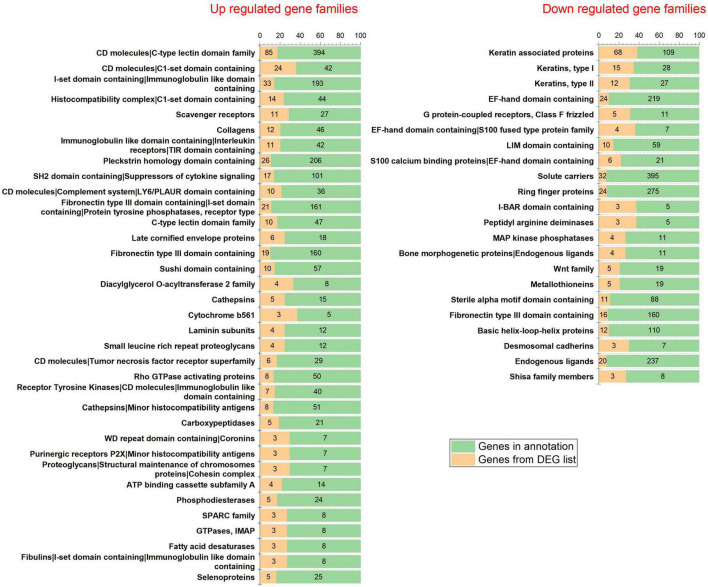
Gene family enrichment analysis of the differentially expressed genes.

The analysis returned a total of 289 DEGs (33 up-regulated and 256 down-regulated DEGs) for a threshold cut off of value log_2_FC > | 1| and *q*-value < 0.05 (Supplementary II-6). Further to construct a big and detailed STRING PPI and reactome FI networks a total of 2,439 unique DEGs (1,261 up-regulated, 1,171 down-regulated, and 7 genes with probes expressed in both directions) that falls within a cut off value of log_2_FC > | 0.3| and *q*-value < 0.05 were mined (Figure 1). The gene family enrichment analysis of these 2,439 DEGs are given in Figure 2 and Supplementary II-7. GO functional analyses revealed that the up-regulated genes enriched for immune system mediated GO terms implying a heightened immune response in hairless scalp, while the down-regulated genes enriched for hair growth related GO terms as expected (Table 1 and Supplementary II-8). The Reactome pathway enrichment analysis also enriched pathways such as keratinization, developmental biology G2/M DNA replication checkpoint for down-regulated genes, wherein for up-regulated genes innate immune system, Cytokine signaling, interferon signaling, adaptive immune system, and antigen processing cross presentation pathways were enriched (Table 2 and Supplementary II-9). The DEG list was inspected for genes known to be involved in various signaling pathways such as Wnt, NF-κB, TGF-β, BMP, and Vitamin D metabolism and the mapped DEGs for these signaling pathways were provided in the Supplementary I-3.

**TABLE 1:** Result of gene ontology analysis of DEGs from ToppGene Suite (FDR < 0.05).

	Gene ontology of up-regulated genes			Gene ontology of down-regulated genes		
	**Gene ontology term**	**Number of genes from DEG list**	**Number of genes in annotation**	**Gene ontology term**	**Number of genes enriched**	**Number of genes in annotation**
Molecular function	Extracellular matrix structural constituent	44	195	Structural constituent of skin epidermis	16	44
	Signaling receptor binding	181	1,813	Structural molecule activity	87	892
	Protein-containing complex binding	166	1,726			
	Oxidoreductase activity	97	834			
	integrin binding	35	171			
	Immune receptor activity	34	165			
	Carbohydrate binding	46	315			
	Antigen binding	32	189			
	MHC protein Complex binding	14	43			
	MHC class II protein complex binding	11	27			
Biological process	Regulation of immune system process	239	1,821	Intermediate filament organization	29	74
	Cell activation	208	1,464	Molting cycle	38	149
	leukocyte activation	184	1,277	Hair cycle	38	149
	Immune effector process	144	895	Intermediate filament cytoskeleton organization	30	96
	Regulation of immune response	159	1,088	Intermediate filament-based process	30	98
	Positive regulation of immune system process	165	1,164	Epithelium development	180	1,979
	Lymphocyte activation	154	1,058	Skin development	60	387
	Cell adhesion	211	1,742	Epidermis development	67	500
	Leukocyte mediated immunity	105	594	Hair follicle development	27	120
	T cell activation	115	704	Hair cycle process	27	123
Cellular component	Cell surface	162	1,178	Intermediate filament	101	229
	External side of plasma membrane	104	599	Keratin filament	73	108
	Side of membrane	124	853	Intermediate filament cytoskeleton	103	271
	Extracellular matrix	106	678	Polymeric cytoskeletal fiber	156	889
	External encapsulating structure	106	680	Supramolecular polymer	177	1,181
	Collagen-containing extracellular matrix	90	541	Supramolecular fiber	176	1,172
	Intrinsic component of plasma membrane	202	1,992	Supramolecular complex	195	1,549
	Integral component of plasma membrane	192	1,893	Anchoring junction	109	1,419
	Secretory granule	116	987	Cell-cell junction	53	590
	MHC protein complex	16	26	Extracellular matrix	58	678

**TABLE 2:** Result of reactome pathway enrichment analysis of DEGs from ToppGene Suite (FDR < 0.05).

Reactome ID	Pathway name	Genes from DEG list	Genes in annotation
**Up-regulated genes**			
1269203	Innate immune system	155	1,302
1269310	Cytokine signaling in immune system	102	760
1270244	Extracellular matrix organization	53	297
1269314	Interferon gamma signaling	27	94
1269201	Immunoregulatory interactions between a Lymphoid and a non-lymphoid cell	31	135
1457780	Neutrophil degranulation	69	492
1269318	Signaling by interleukins	70	528
1269340	Hemostasis	78	639
1269311	Interferon Signaling	33	202
1269173	Phosphorylation of CD3 and TCR zeta chains	10	25
1270246	Collagen biosynthesis and modifying enzymes	17	70
1269171	Adaptive immune system	88	823
1269350	Platelet activation, signaling and aggregation	40	282
1470923	Interleukin-4 and 13 signaling	22	114
1269174	Translocation of ZAP-70 to immunological synapse	9	22
1270260	Integrin cell surface interactions	16	68
1270001	Metabolism of lipids and lipoproteins	84	816
1269182	PD-1 signaling	9	26
1269195	Antigen processing-cross presentation	19	101
1270245	Collagen formation	18	93
**Down-regulated genes**			
1457790	Keratinization	103	214
1270302	Developmental biology	158	1,078
1269756	G2/M DNA replication checkpoint	4	5
1269570	Class B/2 (Secretin family receptors)	16	93

### Annotation of differentially expressed genes with AGA risk loci

Mapping of SNPs identified through GWAS to DEGs annotates genetic variants located in or near the gene regions that are differentially expressed, helps to understand the functional role of DEGs and their association with disease. To identify genes that potentially contribute to AGA pathology, we annotated the coordinates of 107 genomic loci associated with AGA risk in men identified through GWAS with our DEGs. The analysis identified 51 DEGs within the window of 500 kb of 73 AGA risk SNPs and 14 DEGs within the window of 50 kb of 16 lead SNPs (Table 3). Some of the DEGs were mapped to reported AGA risk SNPs including MEMO1 at loci 2p22.3, SRD5A2 at 2p23.1, FOXL2NB at 3q23, FGF5 at 4q21.21, DKK2 at 4q25, EBF1 at 5q33.3, IRF4 at 6p25.3, CENPW at 6q22.32, PAGE2 at Xp11.21, highlighting their association with AGA pathology. Our analysis also identified other DEGs, such as HOXD9 at 2q31.1, LHPP at 10q26.13, CRHR1 at 17q21.31, STH at 17q21.31, and PAGE2B at Xp11.21, as more likely to be candidate gene risks for AGA than the mapped genes in GWAS studies. MEMO1 was down-regulated, and it plays a crucial role in regulating cell proliferation, survival, and differentiation in the hair follicle (30). SRD5A2, whose product inhibits hair growth, was up-regulated (31). HOXD9, a member of the HOX family of genes that plays a crucial role in the development and patterning of various tissues and organs in the body, was up-regulated in our analysis, although its role in hair growth is unknown (32). FGF5, which inhibits hair growth and is involved in the transition of hair follicles from anagen to catagen phase, was down-regulated (33). DKK2, a Wnt inhibitor that leads to hair growth inhibition was up-regulated (1). FOXL2NB, IRF4, CENPW, EBF1, LHPP, CRHR1, and STH located within the AGA risk loci warrant further investigation. The mapped DEGs within 500 kb of lead SNPs may also be considered for future investigation of their association with hair growth and AGA (Table 3).

**TABLE 3:** Overlap of AGA risk loci from genome-wide significance studies with differentially expressed genes in premature AGA samples compared to normal men.

Chr	Cytogenetic region	SNP	Study accession	Mapped genes	500 kb		100 kb		50 kb		Log_2_FC
					**Diff genes**	**Up/Down**	**Diff genes**	**Up/Down**	**Diff genes**	**Up/Down**	
Chr1	1p33	rs61784834	GCST005116	RPL21P24, FOXD2	TRABD2B	Down	–	–	–	–	–0.61
Chr1	1p36.11	rs11249243	GCST005116	RUNX3, MIR4425	RUNX3	Up	RUNX3	Up	–	–	0.38
					CLIC4	Down	–	–	–	–	–0.62
Chr1	1p36.11	rs9803723	GCST005116	IFITM3P7, SYF2	RUNX3	Up	–	–	–	–	0.38
					CLIC4	Down	–	–	–	–	–0.62
Chr1	1p36.11	rs2064251	GCST005116	IFITM3P7, SYF2	RUNX3	Up	–	–	–	–	0.38
					CLIC4	Down	–	–	–	–	–0.62
Chr1	1p36.11	rs7534070	GCST003983	SYF2, IFITM3P7	RUNX3	Up	–	–	–	–	0.38
					CLIC4	Down	–	–	–	–	–0.62
Chr1	1p36.22	rs12565727	GCST001548	C1orf127	ANGPTL7	Down	–	–	–	–	–2.21
Chr1	1p36.22	rs2095921	GCST003983	C1orf127	ANGPTL7	Down	–	–	–	–	–2.21
Chr1	1p36.22	rs7542354	GCST005116	C1orf127	ANGPTL7	Down	–	–	–	–	–2.21
Chr1	1q24.2	rs78003935	GCST003983	GORAB-AS1, HAUS4P1	PRRX1	Up	–	–	–	–	0.54
Chr1	1q24.2	rs11578119	GCST005116	GORAB, GORAB-AS1	PRRX1	Up	–	–	–	–	0.54
Chr2	2p14	rs6546334	GCST003983	LINC01812	CNRIP1	Up	–	–	–	–	0.37
Chr2	2p14	rs62146540	GCST005116	FBXL12P1	CNRIP1	Up	–	–	–	–	0.37
					PLEK	Up	–	–	–	–	0.64
Chr2	2p21	rs11694173	GCST003983	THADA	ZFP36L2	Up	–	–	–	–	0.31
Chr2	2p22.3	rs13021718	GCST005116	DPY30, MEMO1	MEMO1	Down	MEMO1	Down	MEMO1	Down	–0.32
					SRD5A2	Up	–	–	–	–	0.71
Chr2	2p23.1	rs9282858	GCST003983	SRD5A2	SRD5A2	Up	SRD5A2	Up	SRD5A2	Up	0.71
					GALNT14	Down	–	–	–	–	–0.59
					MEMO1	Down	–	–	–	–	–0.32
					EHD3	Down	–	–	–	–	–1.49
					CAPN14	Down	–	–	–	–	–0.92
Chr2	2q13	rs3827760	GCST003983, GCST90043616	EDAR	GCC2	Down	–	–	–	–	–0.35
Chr2	2q31.1	rs13405699	GCST005116, GCST003983	’–	MAP3K20	Down	–	–	–	–	–0.42
Chr2	2q31.1	rs71421546	GCST005116	HOXD-AS2	HOXD9	Up	HOXD9	Up	HOXD9	Up	0.34
Chr2	2q35	rs74333950	GCST003983	WNT10A	CYP27A1	Up	CYP27A1	Up	–	–	0.42
Chr2	2q35	rs7349332	GCST005116	WNT10A	CYP27A1	Up	CYP27A1	Up	–	–	0.42
Chr2	2q37.3	rs9287638	GCST001548	TWIST2, LINC01937	TWIST2	Up	TWIST2	Up	–	–	0.48
Chr2	2q37.3	rs11684254	GCST005116, GCST003983	LINC01937, TWIST2	TWIST2	Up	TWIST2	Up	–	–	0.48
Chr3	3q23	rs6788232	GCST005116	PRR23A, FOXL2NB	FOXL2NB	Up	FOXL2NB	Up	FOXL2NB	Up	0.73
					FOXL2	Up	FOXL2	Up	–	–	0.95
					FOXL2NB	Up	–	–	–	–	0.73
Chr3	3q23	rs7642536	GCST005116, GCST003983	MRPS22	FOXL2	Up	–	–	–	–	0.95
Chr3	3q25.1	rs4679956	GCST003983	AADACL2-AS1	IGSF10	Up	–	–	–	–	0.45
Chr3	3q25.1	rs16863765	GCST005116	AADACL2-AS1	IGSF10	Up	–	–	–	–	0.45
Chr4	4q21.21	rs7680591	GCST005116	FGF5	FGF5	Down	FGF5	Down	FGF5	Down	–1.09
Chr4	4q21.21	rs4690116	GCST003983	FGF5	FGF5	Down	FGF5	Down	FGF5	Down	–1.09
Chr4	4q25	rs78311490	GCST003983	DKK2	DKK2	Up	DKK2	Up	DKK2	Up	0.34
Chr5	5q33.3	rs1422798	GCST005116	EBF1	EBF1	Up	EBF1	Up	EBF1	Up	0.34
					RNF145	Down	–	–	–	–	–0.40
Chr5	5q33.3	rs62385385	GCST003983	EBF1	EBF1	Up	EBF1	Up	EBF1	Up	0.34
					RNF145	Down	–	–	–	–	–0.40
Chr6	6p25.3	rs12203592	GCST005116, GCST003983	IRF4	IRF4	Up	IRF4	Up	IRF4	Up	0.49
Chr6	6q21	rs12214131	GCST005116	’–	PREP	Down	–	–	–	–	–0.37
Chr6	6q22.32	rs9398803	GCST005116	CENPW	CENPW	Down	CENPW	Down	CENPW	Down	–0.32
Chr6	6q22.32	rs1262557	GCST003983	RPS4XP9	CENPW	Down	–	–	–	–	–0.32
Chr7	7p21.1	rs2073963	GCST001548	HDAC9	TWIST1	Up	–	–	–	–	0.57
Chr7	7p21.1	rs71530654	GCST005116	HDAC9	TWIST1	Up	–	–	–	–	0.57
Chr7	7p21.1	rs7801037	GCST003983	HDAC9	TWIST1	Up	–	–	–	–	0.57
Chr7	7q11.22	rs939963	GCST005116	RNU6-832P	AUTS2	Up	–	–	–	–	0.31
Chr7	7q11.22	rs34991987	GCST003983	RNU6-832P	AUTS2	Up	–	–	–	–	0.31
Chr7	7q11.22	rs6945541	GCST001548	RNU6-832P	AUTS2	Up	–	–	–	–	0.31
Chr7	7q11.22	rs4718886	GCST005116	Y_RNA, RNU6-229P	AUTS2	Up	–	–	–	–	0.31
Chr7	7q32.3	rs9719620	GCST005116	MKLN1, MKLN1-AS	LINC-PINT	Up	–	–	–	–	0.40
Chr10	10q22.3	rs11593840	GCST005116, GCST003983	LRMDA	KCNMA1	Up	–	–	–	–	0.46
Chr10	10q26.13	rs3781458	GCST003983	FAM53B	LHPP	Up	LHPP	Up	LHPP	Up	0.30
Chr10	10q26.13	rs3781452	GCST005116	FAM53B	LHPP	Up	LHPP	Up	LHPP	Up	0.30
Chr11	11p11.2	rs11037975	GCST005116, GCST003983	ALX4	CD82	Down	–	–	–	–	–0.33
					ACCS	Up	–	–	–	–	0.56
Chr12	12p11.22	rs7976269	GCST005116	FAR2	TMTC1	Down	–	–	–	–	–0.48
Chr12	12p12.1	rs9300169	GCST003983	SSPN	RASSF8-AS1	Up	–	–	–	–	0.38
Chr12	12p12.1	rs7974900	GCST005116	SSPN	RASSF8-AS1	Up	–	–	–	–	0.38
Chr12	12q13.13	rs180807105	GCST90043616	HOXC12	MAP3K12	Up	–	–	–	–	0.31
					NFE2	Up	–	–	–	–	0.71
Chr12	12q24.33	rs76972608	GCST005116, GCST003983	FZD10-AS1, LINC02419	FZD10	Down	FZD10	Down	–	–	–0.55
Chr13	13q12.3	rs9314998	GCST003983	LINC00385, KATNAL1	LINC00426	Up	–	–	–	–	0.44
Chr17	17q21.31	rs12373124	GCST001548	MAPT-AS1, SPPL2C	CRHR1	Down	CRHR1	Down	CRHR1	Down	–0.72
					STH	Up	STH	Up	STH	Up	0.36
Chr17	17q21.31	rs919462	GCST005116	MAPT	STH	Up	STH	Up	STH	Up	0.36
					CRHR1	Down	–	–	–	–	–0.72
Chr17	17q21.31	rs201408539	GCST003983	KANSL1	STH	Up	STH	Up	–	–	0.36
					CRHR1	Down	–	–	–	–	–0.72
Chr17	17q21.31	rs572756998	GCST005116	ARL17B	CRHR1	Down	–	–	–	–	–0.72
					STH	Up	–	–	–	–	0.36
					WNT3	Down	–	–	–	–	–0.68
Chr17	17q22	rs17833789	GCST005116	AKAP1	MSI2	Down	–	–	–	–	–0.33
					MTVR2	Up	–	–	–	–	0.38
Chr17	17q22	rs62060349	GCST003983	LINC02563, AKAP1	MSI2	Down	–	–	–	–	–0.33
					MTVR2	Up	–	–	–	–	0.38
Chr20	20p11.22	rs2180439	GCST000251, GCST001297	’–	PAX1	Up	–	–	–	–	0.81
Chr20	20p11.22	rs77410716	GCST005116	’–	PAX1	Up	–	–	–	–	0.81
Chr20	20p11.22	rs552649178	GCST005116	LINC01432	PAX1	Up	–	–	–	–	0.81
Chr20	20p11.22	rs201563	GCST003983	LINC01432	PAX1	Up	–	–	–	–	0.81
Chr20	20p11.22	rs6047844	GCST001548	LINC01432	PAX1	Up	–	–	–	–	0.81
Chr20	20p11.22	rs11087368	GCST005116	LINC01432	PAX1	Up	–	–	–	–	0.81
Chr20	20p11.22	rs1160312	GCST000250	LINC01432	PAX1	Up	–	–	–	–	0.81
ChrX	Xp11.21	rs185597083	GCST003983	FAM104B, PAGE2	PAGE2	Up	PAGE2	Up	PAGE2	Up	0.48
ChrX	Xp22.31	rs5933688	GCST003983	ANAPC15P1, NOLC1P1	PAGE2B	Up	PAGE2B	Up	PAGE2B	Up	0.69
					ANOS1	Down	–	–	–	–	–0.61
ChrX	Xp22.31	rs5934505	GCST005116	ANAPC15P1, NOLC1P1	ANOS1	Down	–	–	–	–	–0.61
ChrX	Xq12	rs200644307	GCST003983	’–	AR	Up	–	–	–	–	0.43
ChrX	Xq12	rs6625163	GCST000250	’–	AR	Up	–	–	–	–	0.43
ChrX	Xq12	rs2497938	GCST001548, GCST001297	’-	AR	Up	–	–	–	–	0.43
ChrX	Xq12	rs7061504	GCST005116	OPHN1	AR	Up	–	–	–	–	0.43

### Motif analysis in the promoter regions of AGA differentially expressed genes

**FIGURE 3:**
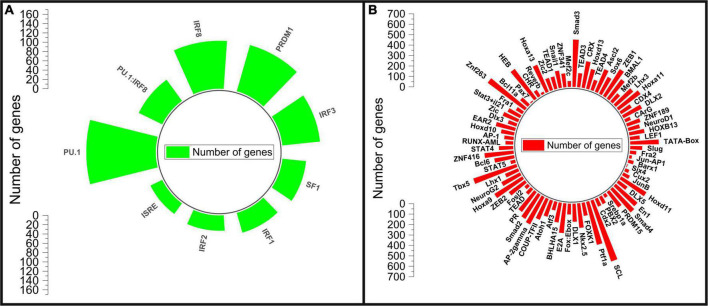
Motif enrichment analysis of the differentially expressed genes. **(A)** Motifs enriched in up-regulated genes, **(B)** motifs enriched in down regulated genes.

The transcription factor motif enrichment analysis on the promoter regions of the differentially expressed genes was carried to identify potential transcription factors involved in the AGA pathology. The top transcription factor motifs enriched for the down-regulated genes are LEF1 (Lymphoid Enhancer binding Factor 1), HOXB13 (Homeobox B13), NEUROD1 (Neuronal Differentiation 1), ZNF189 (Zinc Finger protein 189), and MEF2C (MADS Box Transcription factor 2, Polypeptide C) (Figure 3 and Supplementary II-10). The transcription factor LEF1 actively participates in the Wnt signaling pathway by activating the transcription of target genes in the presence of β-catenin. Wnt/β-catenin Signaling plays a crucial role in hair follicle differentiation and morphogenesis (31). The transcription factor HOXB13 belongs to HOX gene family which plays a crucial role in regulating embryonic development including hair formation. HOXB13 is implicated in skin development and low level of its expression is associated with telogen hair follicle (32, 34). The transcription factor NEUROD1 is primarily involved in the development and differentiation of the nervous system. NEUROD1 acts by controlling the expression of genes involved in neuronal development and in the formation of axons and dendrites (35). ZNF189 belongs to the zinc finger protein family which play important roles in various biological processes including transcriptional regulation, DNA repair, and cellular signaling. MEF2C belongs to the MADS box transcription factor 2 (MEF2) family of transcription factors and is involved in myogenesis (32). Many transcription factor motifs belonging to the SMAD, HOX, STAT, ZNF, NEURO, FOX, and FOS gene families (Figure 3) are enriched for the down-regulated genes indicating their role in hair growth which has to be studied further.

The motifs for IRF3 (Interferon Regulatory Factor 3), PRDM1 (PR/SET Domain 1), IRF8 (Interferon Regulatory Factor 8), SPI1 (Spi-1 Proto-Oncogene), SPI1:IRF8, ISRE (Interferon-sensitive response element), IRF2 (Interferon Regulatory Factor 2), IRF1 (Interferon Regulatory Factor 1) and SF1 transcription factors were enriched as the top motifs for the up-regulated genes (Figure 3 and Supplementary I-11). The Interferon regulatory factors (IRFs) are a family of transcription factors that regulate various aspects of the immune system from promoting immune cell development to immune cell differentiation. They play a central role in controlling the innate and adaptive immune responses to pathogens (33). IRF1 and IRF2 are important in regulating dendritic cells which participates in antigen presentation and bridge the innate and adaptive immune system. IRF3 involves in type I interferon production and IRF8 regulate myeloid cell development (33). PRDM1 coordinates several important functions in the adaptive immune system that support the key effector functions of B and T lymphocytes (36). SPI-1 encodes an ETS-domain transcription factor that control gene expression involving in the development of myeloid and B-lymphoid immune cells (37). The enrichment of these transcription factor motifs as the top motifs in the up-regulated genes of bald scalp implies a state of heightened immune response in AGA.

### STRING protein-protein interaction network analysis and identification of hub genes

**FIGURE 4:**
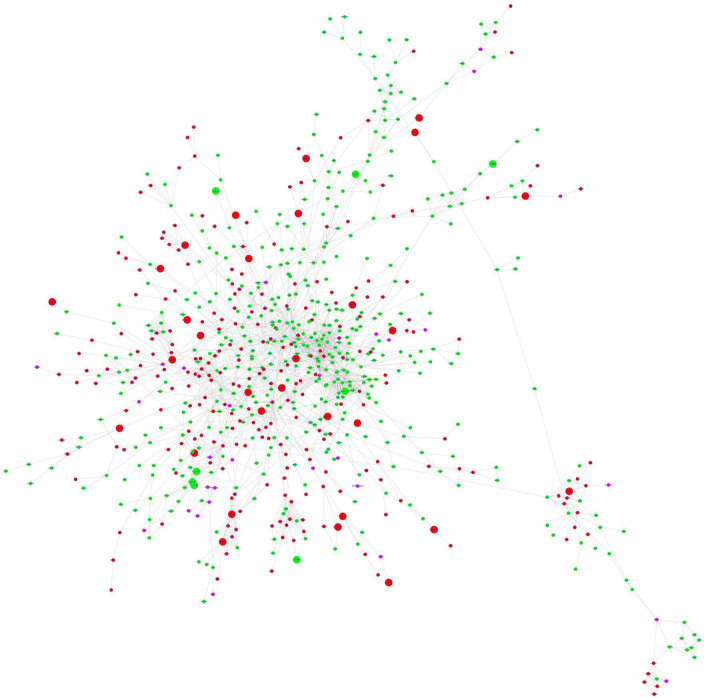
STRING protein-protein interaction network. The nodes are represented as circles and edges as lines. Red and green nodes indicate downregulated and upregulated genes, respectively. The larger nodes represent DEGs that comply with a log_2_FC > | 1| value. The pink nodes indicate linker genes.

The stringApp generated 1967 PPI pairs for the submitted DEGs. The main PPI network, which consisted of 749 nodes (447 up-regulated genes, 273 down-regulated genes, and 29 linker genes) and 1,856 edges, was selected for further analysis while disconnected nodes and small isolated PPI pairs were discarded (Figure 4 and Supplementary II-12). The PPI network had a clustering coefficient of 0.334, a characteristic path length of 5.683, a network diameter of 19, a network density of 0.007, and an average of 4.956 neighbors. The functional enrichment analysis performed using the inbuilt STRING tool on the Reactome and Wikipathway databases revealed that the PPI network was enriched for several immune response-related pathways (Supplementary II-13). The pathway terms related to Cytokine Signaling in the Immune System, Interferon Signaling, T cell receptor signaling, Signaling by Interleukins, Immunoregulatory interactions between a Lymphoid and a non-Lymphoid cell, Adaptive and Innate immune systems, and TCF-dependent signaling in response to WNT were enriched from the Reactome database. Additionally, the Wikipathways database identified significant pathway terms related to the Inflammatory response pathway, Development of pulmonary dendritic cells and macrophage subsets, B cell receptor signaling pathway, and the Vitamin D receptor pathway (Supplementary II-13). These results confirm the credibility of the PPI network and reinforce the observation of immune response-related and hair follicle-related pathways.

The top 20 ranking hub nodes (genes) in the PPI network were identified using the Cytoscape plugin Cytohubba based on four topological analysis methods and two centralities (MCC, DMNC, MNC, Degree, Closeness, and Betweenness) and are listed in Table 4. Out of these, a total of 15 hub genes that appeared in at least three of these categories were considered as significant hub genes, and the frequently appeared genes are highlighted in the Table 4. The MCODE cluster analysis performed on the String PPI network revealed 8 clusters when using the 15 hub genes as roots for clustering. The 8 clusters were comprised of 53, 63, 101, 59, 37, 41, 53, and 9 nodes, respectively (Supplementary II-14). The top 3 highly interconnected clusters were selected for further analysis (Supplementary 1–4). Cluster 1 had 10 hub genes, cluster 2 contained 5 hub genes, and cluster 3 had 6 hub genes. Our analysis revealed that two hub genes LCK and STAT5A appeared in all 3 clusters strongly suggesting their putative role in AGA.

**TABLE 4:** Top 20 hub proteins identified by different topological algorithms and centralities utilizing cytohubba plugin in the STRING PPI network.

Topological algorithms				Centralities	
**MCC**	**MNC**	**DMNC**	**Degree**	**Betweenness**	**Closeness**
PSMB8	LYN	IFITM3	NRAS	CTNNB1	CTNNB1
IRF9	LCK	IFITM1	CTNNB1	SDC1	LCK
ISG15	HLA-A	ISG15	STAT3	COL4A4	STAT3
EGR1	STAT3	IFITM2	LYN	ENPP1	EGF
IFITM3	NRAS	OAS1	LCK	NRAS	LYN
IFITM1	HLA-DRB1	EGR1	HLA-A	HGF	NRAS
IFITM2	PTPN6	IRF8	PTPN6	STAT3	PTPN6
OAS1	STAT5A	HLA-G	STAT5A	PPARA	STAT5A
HLA-A	PSMB8	IRF1	HLA-DRB1	ITGAM	PDGFRB
HLA-F	HLA-F	POFUT2	EGF	PKLR	VAV1
HLA-E	CDK1	SPON1	ITGAM	LYN	HGF
IRF4	HLA-E	THSD4	B2M	H2AX	HCK
IRF1	CTNNB1	ADAMTS7	CDK1	THBS1	ITGAM
IRF8	HCK	ADAMTS1	PSMB8	TNF	ESR1
HLA-G	CCNB1	CFP	HLA-F	EGF	AR
CFP	FGR	ADAMTS17	HLA-E	HIF1A	HIF1A
POFUT2	VAV1	ADAMTS10	CCNB1	ACSL1	CXCL12
SPON1	CCNA2	THBS2	PTPRC	PPARG	HLA-A
THSD4	IRF4	THBS1	VAV1	RACK1	PTPRJ
ADAMTS7	LCP2	IRF9	IRF4	QPRT	SFN

The highlighted genes are present in more than two columns, as indicated by the color code: Violet denotes presence in 4 columns, blue denotes presence in 3 columns, and green denotes presence in 2 columns.

### Reactome protein functional interaction network analysis and identification of hub genes

**FIGURE 5:**
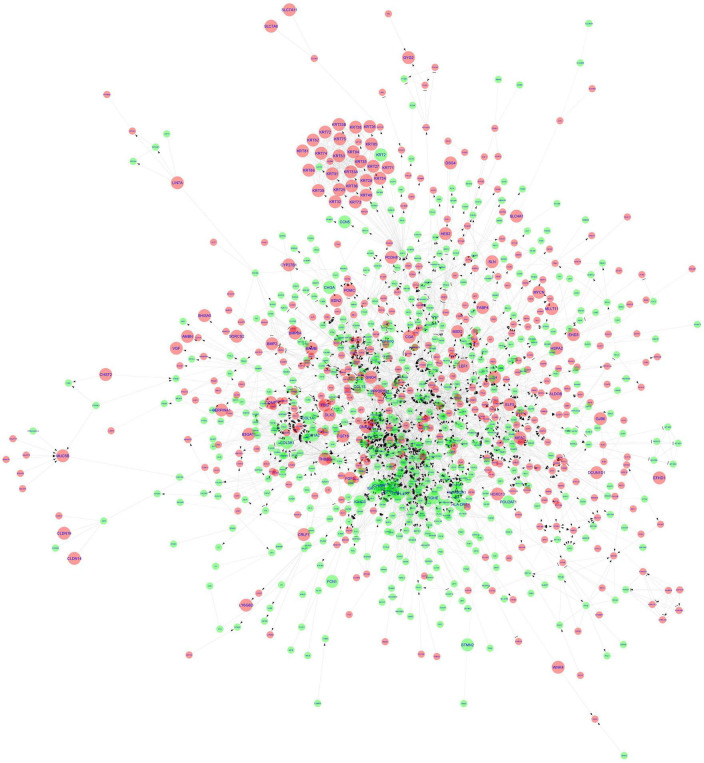
Functional interaction network generated by ReactomeFIViz app. The nodes are represented as circles and edges as lines. Red and green nodes indicate downregulated and upregulated genes, respectively. The larger nodes represent DEGs that comply with a log_2_FC > | 1| value.

The ReactomeFIViz tool was utilized to construct the FI network for the DEGs resulting in an initial network of 1,092 connected nodes, 1,340 unconnected nodes, and 4,047 edges (Supplementary II-15). The unconnected nodes were discarded from the analysis and the final FI network consisted of 1,014 nodes (581 up-regulated and 433 down-regulated genes) with 3,980 edges as shown in Figure 5. The FI network had a clustering coefficient of 0.280, a network diameter of 11, a network density of 0.008, and an average number of neighbors of 7.850. The pathway enrichment and GO Biological process analyses were conducted using the inbuilt ReactomeFIViz – analysis network function tool (Supplementary II-16). Reactome pathway terms such as Extracellular matrix organization, Keratinization, Interferon gamma signaling, Regulation of Insulin-like Growth Factor (IGF) transport and uptake by Insulin-like Growth Factor Binding Proteins, Response to elevated platelet cytosolic Ca2 +, Immunoregulatory interactions between a Lymphoid and a non-Lymphoid cell, and WNT ligand biogenesis and trafficking were enriched for the genes present in the FI network. GO Biological process terms such as immune response, transmembrane receptor protein tyrosine kinase signaling pathway, canonical Wnt signaling pathway, and inflammatory response were also enriched.

The top 20 ranked hub genes in the FI network identified based on the six algorithms including MCC, DMNC, MNC, Degree, Closeness, and Betweenness are presented in the Table 5. Out of these, a total of 19 hub genes that appeared in at least three of the categories were considered significant hub genes, and the frequently appeared genes are highlighted in the Table 5. The MCODE cluster analysis of the FI network revealed 11 clusters when using the 19 hub genes as roots for clustering. The 11 clusters had node numbers of 189, 55, 216, 47, 34, 59, 153, 180, 29, 69, and 6, respectively (Supplementary II-17). The top 3 clusters ranked based on their cluster score were selected for further analysis (Supplementary I-5). Cluster 1 consisted of 14 hub genes, cluster 2 contained 1 hub genes, and cluster 3 had 9 hub genes. Our results showed that seven hub genes (HCK, GNAI3, RAC2, PDGFRB, EGF, NRAS, and STAT5A) were present in two of the selected clusters indicating their potential role in AGA.

**TABLE 5:** Top 20 hub proteins identified by different topological algorithms and centralities utilizing cytohubba plugin in the reactome FI network.

Topological algorithms				Centralities	
**MCC**	**MNC**	**DMNC**	**Degree**	**Betweenness**	**Closeness**
LCP2	STAT3	LAT2	STAT3	ESR1	STAT3
HLA-DRB1	LYN	HLA-DOA	CTNNB1	CTNNB1	CTNNB1
HLA-DPA1	CTNNB1	CD74	SPI1	STAT3	PIK3CD
HLA-DRB4	LCK	SGO1	ESR1	SPI1	PTPN6
HLA-DQA1	PIK3CD	SKA2	PIK3CD	PIK3CD	SPI1
HLA-DRB3	SPI1	ZWINT	LYN	GNAI3	LYN
HLA-DPB1	PTPN6	C1R	LCK	PTPN6	ESR1
LCK	NRAS	C1S	NRAS	NRAS	STAT5A
CD3E	GNAI3	LCP1	GNAI3	HIF1A	LCK
ZAP70	VAV1	CIITA	PTPN6	EGR1	NRAS
IGKC	HCK	KIF26A	RAC2	ITGB7	EGF
IGKV1-16	EGF	C2	HCK	GATA2	GNAI3
IGLV1-44	PDGFRB	CELSR1	VAV1	AR	HCK
IGLV1-47	LCP2	PCDHB4	EGF	LYN	PDGFRB
IGKV1D-16	RAC2	PCDH7	STAT5A	RAC2	HIF1A
LYN	STAT5A	PCDH8	PDGFRB	TGFBR2	EGR1
VAV1	BTK	DCHS1	ITGB7	ITGAM	AR
BTK	ITGB7	KIF4A	LCP2	TNF	VAV1
HLA-DMB	CD3E	CDH23	ITGAM	STAT5A	CRKL
HLA-DOA	HLA-DRB1	PCDH11Y	BTK	LCK	BTK

The highlighted genes are present in more than two columns, as indicated by the color code: Red denotes presence in 5 columns, violet denotes presence in 4 columns, blue denotes presence in 3 columns, and green denotes presence in 2 columns.

### Candidate genes in AGA pathology

The 25 hub genes identified from the analyses of the PPI and FI networks constructed based on the DEGs were considered key genes in the pathology of AGA (Table 6). Out of these 25 hub genes, 21 genes (BTK, ESR1, HCK, ITGB7, LCK, LCP2, LYN, PDGFRB, PIK3CD, PTPN6, RAC2, SPI1, STAT3, STAT5A, VAV1, PSMB8, HLA-A, HLA-F, HLA-E, IRF4, and ITGAM) were found to be up-regulated, while 4 genes (CTNNB1, EGF, GNAI3, and NRAS) were downregulated. The results of the GO biological process and pathway enrichment analysis, conducted using the Toppgene suite, revealed that the hub genes were associated with immune and inflammatory processes (Table 7). The significant biological terms enriched for the hub genes included regulation of immune system process, T cell activation, immune response-regulating cell surface receptor signaling pathways. Furthermore, the significant pathway terms enriched for the hub genes included cytokine signaling in the immune system, signaling by interleukins, signaling by the B cell receptor (BCR), and signaling by SCF-KIT, interleukin-3, 5, and GM-CSF signaling, and the innate immune system.

**TABLE 6:** Hub genes identified common in the STRING PPI and reactome FI network.

Sr. no.	Gene symbol	Gene name	Gene family	Gene function summary from Uniprot	Expression direction
1	CTNNB1	Catenin beta 1	Armadillo family of proteins	Important for Wnt signaling and cell adhesion	DOWN
2	EGF	Epidermal growth factor	Epidermal growth factor family	Important for cell growth and differentiation	DOWN
3	GNAI3	Guanine nucleotide-binding protein G(I) Subunit Alpha-3	G protein alpha inhibitory subunit family	Regulates diverse signaling pathways	DOWN
4	NRAS	NRAS proto-oncogene, Gtpase	Ras family of small GTPases	Involves in regulating cell growth and differentiation	DOWN
5	BTK	Bruton tyrosine kinase	Tec family of non-receptor tyrosine kinases	Critical for B cell development and activation	UP
6	ESR1	Estrogen receptor 1	Nuclear receptor family	Acts as a transcription factor for estrogen signaling	UP
7	HCK	HCK proto-oncogene, Src family tyrosine kinase	Src family of non-receptor tyrosine kinases	Has roles in immune cell signaling and activation	UP
8	ITGB7	Integrin subunit beta 7	Integrin family of cell adhesion molecules	Important for immune cell trafficking and activation	UP
9	LCK	LCK proto-oncogene, Src family tyrosine kinase	Src family of non-receptor tyrosine kinases	Plays a critical role in T cell development and activation.	UP
10	LCP2	Lymphocyte cytosolic protein 2	SLP-76 family of adapter proteins	Essential for T cell receptor signaling and activation	UP
11	LYN	LYN proto-oncogene, Src family tyrosine kinase	Src family of non-receptor tyrosine kinases	Functions in B cell signaling and immune responses.	UP
12	PDGFRB	Platelet derived growth factor receptor beta	Rho family of small GTPases	Involves in actin cytoskeleton organization and cell migration	UP
13	PIK3CD	Phosphatidylinositol-4,5-bisphosphate 3-kinase Catalytic Subunit Delta	Phosphoinositide 3-kinase catalytic subunit family	Plays a role in various signaling pathways	UP
14	PTPN6	Protein tyrosine phosphatase non-receptor type 6	Protein tyrosine phosphatase family	Regulates immune cell signaling and homeostasis	UP
15	RAC2	Rac family small Gtpase 2	Rho family of small GTPases	Involves in actin cytoskeleton organization and cell migration	UP
16	SPI1	Spi-1 proto-oncogene	ETS family of transcription factors	Essential for hematopoietic development and differentiation	UP
17	STAT3	Signal transducer and activator Of transcription 3	STAT family of transcription factors	Involves in cytokine signaling and immune responses	UP
18	STAT5A	Signal transducer and activator Of transcription 5A	STAT family of transcription factors	Important for immune cell development and activation	UP
19	VAV1	Vav guanine nucleotide exchange factor 1	Vav family of guanine nucleotide exchange factors	Regulates signaling pathways downstream of receptors	UP
20	PSMB8	Proteasome 20s subunit beta 8	Proteasome beta subunit family	Involves in protein degradation and antigen presentation	UP
21	HLA-A	Major histocompatibility complex, Class I, A	Human leukocyte antigen (HLA) family	Involves in antigen presentation and immune responses	UP
22	HLA-F	Major histocompatibility complex, class I, F	Human leukocyte antigen (HLA) family	Involves in immune tolerance and immune responses	UP
23	HLA-E	Major histocompatibility complex, class I, E	Human leukocyte antigen (HLA) family	Involves in antigen presentation and immune regulation	UP
24	IRF4	Interferon regulatory factor 4	Interferon regulatory factor family	Involves in immune cell differentiation and function	UP
25	ITGAM	Integrin subunit alpha M	Integrin family of cell adhesion molecules	Important for leukocyte function and immune responses	UP

**TABLE 7:** Result of GO biological process and reactome pathway enrichment analysis of hub genes from ToppGene Suite (FDR < 0.05).

GO ID	Biological process term	Gene count	Up-regulated genes	Down-regulated genes
GO:0002682	Regulation of immune system process	20	IRF4, PTPN6, LCK, SPI1, HLA-A, LCP2, LYN, ITGAM, PIK3CD, HCK, VAV1, ESR1, STAT3, BTK, STAT5A, RAC2, HLA-E, HLA-F	CTNNB1, NRAS
GO:0042110	T cell activation	15	IRF4, PTPN6, LCK, SPI1, HLA-A, LYN, ITGAM, PIK3CD, VAV1, STAT3, STAT5A, RAC2, HLA-E, HLA-F	CTNNB1
GO:0050778	Positive regulation of immune response	15	PTPN6, LCK, SPI1, HLA-A, LCP2, LYN, ITGAM, PIK3CD, HCK, VAV1, BTK, STAT5A, HLA-E, HLA-F	NRAS
GO:0043299	Leukocyte degranulation	10	SPI1, HLA-A, LYN, ITGAM, PIK3CD, HCK, BTK, RAC2, HLA-E, HLA-F	
GO:0002764	Immune response-regulating signaling pathway	14	IRF4, PTPN6, LCK, HLA-A, LCP2, LYN, PIK3CD, HCK, VAV1, ESR1, BTK, HLA-E, HLA-F	NRAS
GO:1903131	Mononuclear cell differentiation	14	IRF4, PTPN6, LCK, SPI1, LYN, PIK3CD, VAV1, STAT3, BTK, STAT5A, RAC2, HLA-E, HLA-F	CTNNB1
GO:0045321	Leukocyte activation	17	IRF4, PTPN6, LCK, SPI1, HLA-A, LCP2, LYN, ITGAM, PIK3CD, VAV1, STAT3, BTK, STAT5A, RAC2, HLA-E, HLA-F	CTNNB1
GO:0002521	Leukocyte differentiation	15	IRF4, PTPN6, LCK, SPI1, LYN, ITGAM, PIK3CD, VAV1, STAT3, BTK, STAT5A, RAC2, HLA-E, HLA-F	CTNNB1
GO:0046649	Lymphocyte activation	16	IRF4, PTPN6, LCK, SPI1, HLA-A, LYN, ITGAM, PIK3CD, VAV1, STAT3, BTK, STAT5A, RAC2, HLA-E, HLA-F	CTNNB1
GO:0002768	Immune response-regulating cell surface receptor signaling pathway	12	PTPN6, LCK, HLA-A, LCP2, LYN, PIK3CD, HCK, VAV1, BTK, HLA-E, HLA-F	NRAS
**Biosystems ID**	**Reactome pathway name**	**Gene count**	**Up-regulated genes**	**Down-regulated genes**
1269310	Cytokine signaling in immune system	17	PSMB8, IRF4, PTPN6, LCK, HLA-A, LYN, ITGAM, PDGFRB, PIK3CD, HCK, VAV1, STAT3, STAT5A, HLA-E, HLA-F	NRAS, EGF
1269318	Signaling by interleukins	14	PSMB8, IRF4, PTPN6, LCK, LYN, ITGAM, PDGFRB, PIK3CD, HCK, VAV1, STAT3, STAT5A	NRAS, EGF
1269171	Adaptive immune system	15	PSMB8, PTPN6, LCK, HLA-A, LCP2, LYN, PDGFRB, PIK3CD, ITGB7, VAV1, BTK, HLA-E, HLA-F	NRAS, EGF
1269357	GPVI-mediated activation cascade	7	PTPN6, LCK, LCP2, LYN, PIK3CD, VAV1, RAC2	
1269183	Signaling by the B cell receptor (BCR)	10	PSMB8, PTPN6, LCK, LYN, PDGFRB, PIK3CD, VAV1, BTK	NRAS, EGF
1269487	Signaling by SCF-KIT	11	PSMB8, PTPN6, LCK, LYN, PDGFRB, PIK3CD, VAV1, STAT3, STAT5A	NRAS, EGF
1269323	Interleukin-3, 5 and GM-CSF signaling	10	PSMB8, PTPN6, LYN, PDGFRB, PIK3CD, HCK, VAV1, STAT5A	NRAS, EGF
1269203	Innate immune system	16	PSMB8, PTPN6, LCK, HLA-A, LCP2, LYN, ITGAM, PDGFRB, PIK3CD, HCK, VAV1, BTK, HLA-E	NRAS, EGF
1269284	DAP12 signaling	10	PSMB8, LCK, LCP2, PDGFRB, PIK3CD, VAV1, BTK, HLA-E	NRAS, EGF
1268855	Diseases of signal transduction	10	PSMB8, LCK, PDGFRB, PIK3CD, VAV1, STAT3, STAT5A	CTNNB1, NRAS, EGF

CTNNB1 (Catenin Beta 1) is a crucial downstream component of the Canonical Wnt Signaling Pathway. In the presence of Wnt ligand, β-catenin accumulates in the nucleus and functions as a coactivator for the transcription factors TCF/LEF, leading to the activation of Wnt responsive genes (35). The Wnt/β-catenin signaling pathway is essential for hair growth and its inhibition, driven by 5α-dihydrotestosterone through the androgen receptor, can result in hair loss in AGA (1). GNAI3 (G Protein Subunit Alpha I3) functions as a downstream transducer of G protein-coupled receptors (GPCRs) in various signaling pathways (38). GPCRs play a role in regulating skin homeostasis and maintaining hair growth (39–41). NRAS (NRAS Proto-Oncogene, GTPase) is a membrane protein that travels between the plasma membrane and Golgi apparatus (42). EGF (Epidermal Growth Factor) acts as a switch in the hair growth cycle (43). It regulates the expression of hair follicle regulatory genes through Wnt//β-catenin signaling (44). Thus, these 4 downregulated hub genes which are involved in hair growth mechanisms are crucial and their downregulation in AGA is expected.

The LCK (LCK Proto-Oncogene, Src Family Tyrosine Kinase) gene, which encodes a non-receptor protein-tyrosine kinase, is a crucial signaling molecule in the selection and maturation of developing T cells and plays a key role in T cell receptor signal transduction pathways (25, 26). The up-regulation of the LCK gene is also associated with alopecia areata (27). The LYN (LYN Proto-Oncogene, Src Family Tyrosine Kinase) gene encodes a non-receptor tyrosine-protein kinase and is crucial for regulating innate and adaptive immune responses, integrin signaling, growth factor and cytokine responses, and hematopoiesis (24). BTK (Bruton Tyrosine Kinase) and plays a key role in B lymphocyte development and is a target for inflammatory diseases (45). Inhibition of BTK by inhibitors leads to changes in hair and nails texture (38). PIK3CD (Phosphatidylinositol-4,5-Bisphosphate 3-Kinase Catalytic Subunit Delta) is involved in immune system response (46). PTPN6 (Protein Tyrosine Phosphatase Non-Receptor Type 6) is critical for the function of lymphoid and myeloid cells (47). SPI1 (Spi-1 Proto-Oncogene) encodes a transcriptional activator specifically involved in the development of macrophages and B cells. This protein also regulates pre-mRNA splicing (23). STAT3 (Signal Transducer and Activator of Transcription 3) is activated by cytokines and growth factors. This gene plays an important role in maintaining the homeosis of skin (48). STAT5A (Signal Transducer and Activator of Transcription 5A) protein serves a dual function of signal transduction and activation of transcription in cells exposed to cytokine and other growth factors. This protein also mediates cellular responses to activated FGFR1, FGFR2, FGFR3 and FGFR4 (31). Also, STAT5 activation is important for hair growth phase induction in hair dermal papilla cells (DPCs) (34). VAV1 (Vav Guanine Nucleotide Exchange Factor 1) encoded protein is important in hematopoiesis and plays a role in the development and activation of T-cell and B-cell (32). PSMB8 (Proteasome 20S Subunit Beta 8) plays an important role in cellular homeostasis through selective destruction of ubiquitinated proteins. Mutations in this gene are associated with autoinflammatory responses (49). ESR1 (Estrogen Receptor 1) is a nuclear sex steroid hormone receptor which regulates many genes responsible for growth, metabolism and reproductive functions. This gene is known to express in hair follicle cells (50). HCK (HCK Proto-Oncogene, Src Family Tyrosine Kinase) participates in the regulation of innate immune responses by inducing monocyte, neutrophil, macrophage and mast cell functions. This gene is recently reported to play a role in hair regenerative potential of stem cells (51). ITGB7 (Integrin Subunit Beta 7) is an adhesion receptor which mediates signaling from the extra cellular matrix to the cell. They also function as a homing receptor for lymphocytes migration (46). LCP2 (Lymphocyte Cytosolic Protein 2) acts as a substrate for the T cell antigen receptor mediated intracellular tyrosine kinase pathway (46). PDGFRB (Platelet Derived Growth Factor Receptor Beta) gene encodes a cell surface tyrosine-protein kinase receptor for the members of the platelet-derived growth factor family. It plays an essential role in cell proliferation, differentiation, survival, chemotaxis, and migration (52). RAC2 (Rac Family Small GTPase 2) involve in phagocytosis of apoptotic cells and epithelial cell polarization (46). IRF4 (Interferon Regulatory Factor 4) regulates interferon signaling and negatively regulates Toll like receptor in the induction of innate and adaptive immune systems (42). ITGAM (Integrin Subunit Alpha M) functions as macrophage receptor and plays a key role in the adherence of monocytes and neutrophils (42). HLA-A (Major Histocompatibility Complex, Class I, A), HLA-F (Major Histocompatibility Complex, Class I, F) and HLA-E (Major Histocompatibility Complex, Class I, E) plays a central role in immune system by participating in cell presentation for recognition by T cell receptor (42). A majority of the hub genes namely PTPN6, LCK, LCP2, LYN, HCK, VAV1, STAT3, STAT5A, and BTK belongs to the Src homology 2 (SH2) domain containing tyrosine kinases and participate in the immune system process.

**FIGURE 6:**
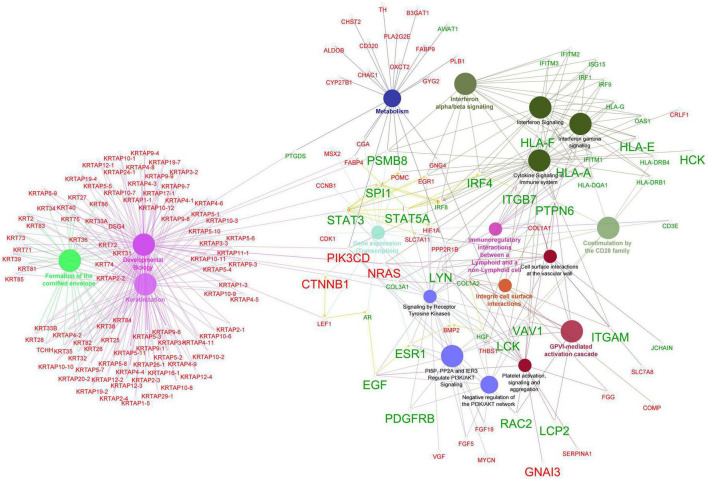
Enrichment of reactome pathway terms for the hub genes and DEGs that comply log_2_FC > | 1| using Cytohubba plugin ClueGO. The network shows the connectivity between pathway terms based on shared functional nodes and edges among the DEGs with a kappa score of 0.4. The color of the nodes and edges represents their specific functional classes, and only significant values with *p* < 0.05 are shown in the enrichment. Nodes labeled in red represent down-regulated genes, while nodes labeled in green indicate up-regulated genes. The larger labeled nodes are the hub genes.

We conducted ClueGO reactome pathway enrichment analysis for the genes that were identified by at least two algorithms of the Cytohubba analysis of the biological networks (PPI and FI) as well as 289 DEGs that met the cut-off value of log_2_FC > | 1| using the ClueGo plugin v2.5.9 in Cytoscape (53). The results were presented as a network of pathways with genes participating in the pathways, which are illustrated in Figure 6. The analysis revealed pathways such as keratinization, formation of the cornified envelope, developmental biology, interferon alpha/beta signaling, cytokine signaling in the immune system, receptor tyrosine kinase signaling, PI5P, PP2A, and IER3 regulation of PI3K/AKT signaling, immunoregulatory interactions between lymphoid and non-lymphoid cells, costimulation by the CD28 family, and the GPVI-mediated activation cascade are the predominant pathways for our input genes. The enrichment of pathways involved in immune system function are consistent with our findings suggesting that immune system dysregulation plays a role in AGA pathology.

### Validation of DEGs with other datasets

To validate the results of our analysis of the GEO dataset GSE90594, we compared the DEGs obtained with other datasets available in the GEO database. As of November 1, 2022, we found that no profile in the database contained samples from men with AGA and from normal haired men, except for the profile we analyzed in this study. The few available datasets related to AGA lacked control samples from normal men and the quality of the microarray and RNA-Seq data was questionable. Despite these limitations, we selected two datasets, GSE66663 (which includes hTERT-immortalized DPCs derived from balding frontal and non-balding occipital scalp samples from men with AGA) and GSE212301 (which contains RNA-Seq data from balding vertex and non-balding occipital scalp samples of 10 men with AGA), and performed differential gene expression analyses. The common DEGs between the datasets are presented in the Supplementary II-18. We discovered 490 DEGs were common between GSE90594 and GSE66663 dataset in which 190 genes were differentially regulated in same directions. Whereas 180 DEGs were common between GSE90594 and GSE212301 dataset in which 44 genes were differentially regulated in same directions.

## Discussion

Differential gene expression analysis is a technique used to identify genes whose expression levels change significantly between two or more experimental conditions or samples using the data generated from microarray or RNA sequencing experiments. This approach helps to determine which genes are upregulated or downregulated in response to a specific condition, such as a disease state or treatment, which facilitates understanding of the underlying molecular mechanisms of diseases (54). In this study we analyzed gene expression data from the scalps of 9 individuals with premature AGA and 10 normal volunteers from the GEO database profile GSE90594 to identify core genes associated with AGA (5). In Michel et al. (5) analysis report, the authors performed differential gene expression analysis on all 28 samples (14 alopecia and 14 normal samples) using ANOVA and Tukey’s *post-hoc* tests. After applying the Benjamini-Hochberg correction for multiple testing, they identified 333 DEGs consisting of 184 downregulated and 149 upregulated genes. The authors selected the DEGs using a cut-off of fold change ≥ ± 1.5 (log_2_FC ≥ ± 0.58) and *p* ≤ 0.05 for significance (5). In our analysis, we normalized the microarrays, removed the outlier samples, and performed the differential gene expression analysis using the single-channel design matrix provided in the *limma* package. We used Benjamini and Hochberg’s method to compute the adjusted *p*-values (FDR or *q*-value) and considered probes with *q* ≤ 0.05 to be significant. In our analysis, the fold change values of AGA-associated genes known to play a crucial role in disease pathology, such as AR (log_2_FC = 0.33), CTNNB1 (log_2_FC = −0.58), TGFB2 (log_2_FC = −0.58), and SRD5A2 (log_2_FC = 0.56) between the AGA patients and healthy group were lower. To thoroughly examine the pathology of AGA, we adopted a stringent criterion of log_2_FC ≥ ± 0.3 with a strict FDR value (*q* ≤ 0.05) and obtained 2,439 DEGs, taking into account that subtle differences in gene expression can have a significant biological impact and that some genes are more sensitive to changes in dosage (55, 56).

To shed light on the biological roles and processes associated with the 2,439 DEGs, we performed gene family enrichment, GO (biological process, molecular function, and cellular component) enrichment, and pathway enrichment analyses. Our results revealed that the down-regulated genes belonged to gene families such as keratins, keratin-associated proteins, frizzled receptors, Bone morphogenetic proteins, Wnt, and metallothioneins (Figure 2). The GO enrichment analysis indicated that these down-regulated genes play vital roles in the structural constituents of the skin epidermis, hair follicle development and hair cycle (Table 1). The pathway enrichment analysis showed that these down-regulated genes participate in the keratinization pathway (Table 2). On the other hand, the up-regulated genes were enriched for CD molecules, Immunoglobulin-like domains, Rho GTPase-activating proteins, receptor tyrosine kinases, minor histocompatibility antigens, and selenoproteins as the top gene families (Figure 2). The GO enrichment analysis also demonstrated that these up-regulated genes were involved in MHC protein complex binding, leukocyte activation, regulation of the immune response, and T-cell activation (Table 1). The pathway enrichment analysis found that the up-regulated genes participated in the innate and adaptive immune systems, cytokine signaling, and interferon signaling pathways (Table 2).

The identification of genetic variants associated with AGA is critical for understanding its etiology. In this study, we annotated the coordinates of AGA-associated genomic loci with our DEGs to identify the potential candidate genes contributing to AGA pathology. Our analysis identified several DEGs located within or near reported AGA risk loci such as MEMO1, SRD5A2, FOXL2NB, FGF5, DKK2, EBF1, IRF4, CENPW, and PAGE2. These findings support the existing knowledge of the association between these genes and AGA pathology. Furthermore, our analysis (Table 3) mapped several DEGs including HOXD9, LHPP, CRHR1, STH, and PAGE2B, which are of unknown significance in hair growth, with AGA risk loci in GWAS studies. These genes warrant further investigation. Moreover, the enrichment of many DEGs identified in our analysis within the 500 kb window of AGA risk loci revealed that the genes which have not yet been identified as AGA risk loci could play a critical role in AGA pathology.

In our analysis to identify specific sequence motifs or patterns in the promoter regions of the DEGs, we found several enriched motifs for the down-regulated genes, including those involved in the Wnt/β-catenin signaling pathway (LEF1), TGF-β signaling (SMAD2, SMAD3, and SMAD4), nervous system development (NeuroD1 and NeuroG2), development (HOXB13, HOXD10, HOXA13, HOXA11, HOXD11, and HOXD13), Jun/FOS family (JunB, Jun-AP1, AP-2 gamma, Fosl2, and AP-1), and FOX family (FOXK1, and Fox:Ebox). Among the down-regulated DEGs, we observed the presence of LEF1, SMAD6, SAMD7, HOXA3, HOXC13, FOXN1, FOXE1, and FOXI2. In contrast, the transcription factors such as NEUROD2, HOXD1, HOXD9, FOXL2, and FOXL2NB were up-regulated, confirming that the down-regulation of hair-related genes in AGA may be primarily due to the Wnt/β-catenin signaling component LEF1 (1).

Furthermore, our motif analysis revealed that the top motifs enriched for the up-regulated genes were those for immune system-related transcription factors, such as IRF1, IRF2, IRF3, IRF8, PRDM1, SPI-1 (PU.1), and SF1. Among the up-regulated DEGs, we observed the presence of several IRF family of transcription factors, including IRF1, IRF1-AS1, IRF4, IRF8, IRF9, and SPI. Specifically, IRF1 is critical for apoptosis and the target genes of IRF1 are responsible for apoptotic responses. IRF4 and IRF8 regulate myeloid cell development, while IRF9 mediates STAT1/STAT2 function in downstream signaling of type I IFN receptor signaling and is also involved in autoantibody production (35, 55). These findings suggest that these immune transcription factors may play a role in the up-regulation of immune response genes, implying a heightened immune system activity and immune response against hair growth cycle in the scalp in AGA. Taken together, our results provide further evidence that the genes for hair follicle development and hair cycle are down-regulated, while genes for immune response are up-regulated in the balding scalps of AGA.

The occurrence of inflammatory phenomena in AGA pathogenesis has been reported earlier, but the cause of the inflammation was unknown. Consequently, the role of inflammation in AGA was not heavily emphasized in the past (52, 57). Despite the general belief that scalp inflammation results in folliculitis, perifollicular fibrosis, and destructive scarring alopecia, studies have linked inflammation to male pattern baldness (55–57). Jaworsky et al. (58) discovered the presence of activated T-cell infiltrate in hair follicles and found that these infiltrates were associated with class II antigens. In 2001, Young et al. (56) discovered granular immunoglobulin M and C3 at the basement membrane, as well as porphyrins in the pilosebaceous canal in biopsy specimens from the bald scalps of AGA patients. They suggested that the local microbiologic flora and environmental factors like UV light could be responsible for the inflammatory reactions (56). Mahe et al. (59) proposed in a 2001 review that the inflammatory process associated with AGA be referred to as microinflammation in contrast to classical inflammatory process. Furthermore, the presence of peripilar signs around the hair follicle ostium, which reflect perifollicular inflammation, has established the presence of follicular microinflammation in AGA (60, 61). Despite these findings, the underlying biological reason, pathways, and genes involved in the inflammatory process of AGA have not yet been elucidated.

In order to deepen our understanding of the inflammatory mechanisms in AGA, we constructed gene interaction networks using the DEGs identified in our study. The Cytoscape plugins StringApp and ReactomeFIplugin were utilized to construct the PPI and FI networks, respectively. The DEGS in the PPI network were connected based on their protein-protein interactions obtained from the STRING database, while the DEGs in the FI network were linked based on their involvement in signaling pathways from the Reactome database. The integrated tools within the Cytoscape StringApp and ReactomeFI plugins were utilized to perform GO and pathway enrichment analyses for both networks. The results were consistent with our previous GO, pathway, and motif enrichment analyses. In addition, a Cytohubba analysis was conducted to identify the hub genes of the biological networks. The hub genes were sorted based on their occurrence in more than one algorithm used in the analysis. As a result, 15 genes (LYN, HLA-A, STAT3, NRAS, CTNNB1, PSMB8, HLA-F, HLA-E, IRF4, LCK, PTPN6, STAT5A, VAV1, EGF, and ITGAM) were identified as key hub genes in the PPI network. Similarly, 19 genes (LCK, LYN, BTK, CTNNB1, GNAI3, NRAS, PIK3CD, PTPN6, SPI1, STAT3, STAT5A, VAV1, EGF, ESR1, HCK, ITGB7, LCP2, PDGFRB, and RAC2) were recognized as key hub genes in the FI network as they were consistently identified across multiple algorithms.

To explore the connections between hub genes and DEGs exhibiting log_2_FC > | 1|, we performed reactome pathway enrichment analysis using the ClueGo plugin in Cytoscape (53). The analysis revealed that the hub genes were strongly associated with several important pathways related to immune system functions including interferon signaling, cytokine signaling, GPVI-mediated activation cascade, PI3K/AKT signaling, and signaling by receptor tyrosine kinases (Figure 6). Interestingly, recent research has linked the activation of the PI3K/Akt pathway with the apoptosis of hair follicle stem cell (HFSC) mediated by 5α-DHT in AGA (62, 63). Furthermore, the ClueGo network (Figure 6) showed that several genes including the hub genes PSMB8, SPI1, STAT3, PIK3CD, NRAS, CTNNB1, and LEF1 connect the keratinization process with inflammatory process terms suggesting that AGA is driven by a complex interplay between various molecular pathways involving immune system dysregulation and abnormal keratinization.

A significant number of the up-regulated hub genes identified in our study such as PTPN6, LCK, LCP2, LYN, HCK, VAV1, STAT3, BTK, and STAT5A belong to the Src Homology 2 (SH2) domain gene family. This group of genes encodes proteins containing SH2 domains, which can recognize and bind to phosphorylated tyrosine residues in other proteins. SH2 domain-containing proteins participate in signal transduction pathways serving as adapter molecules linking tyrosine phosphorylation events to downstream signaling pathways (64). Further of the four non-receptor tyrosine kinase hub genes (BTK, HCK, LCK and LYN), three genes namely HCK, LCK, and LYN belong to the Src family of protein tyrosine kinases (65). Recent studies have highlighted the potential role of Src tyrosine kinase in hair growth. One study found that Src inhibition promotes melanogenesis, leading to the production of hair color pigment melanin (66). In another study the flavonoid quercitrin was shown to stimulate hair growth in cultured DPCs by activating several signal transduction elements, including receptor tyrosine kinases and non-receptor tyrosine kinases. Specifically, Src family proteins such as CSK, FRK, HCK, and SRMS, which were not differentially expressed in our analysis, were found to be activated by quercitrin while promoting the hair growth (67). Additionally, recent researches have shown that Src tyrosine kinase can cross-talk with Wnt signaling (65) and with androgen receptor (AR) signaling (66) suggesting a potential interplay between Src tyrosine kinase and androgen-DHT and Wnt/β-catenin signaling in the balding scalps of AGA. Therefore, we suggest that further investigation into the potential interactions between Src tyrosine kinase family genes, AR-5α-DHT, Wnt/β-catenin signaling, and the inflammatory response is needed to gain a more comprehensive understanding of AGA pathogenesis.

The Hair follicle is a fascinating mini-organ that continuously undergoes cycles of growth (anagen), regression (catagen), resting (telogen), and shedding (exogen). This process is regulated by a number of signaling cascades, including Wnt/β-catenin, Sonic Hedgehog (SHH), bone morphogenetic protein (BMP), notch, transforming growth factor β (TGF-β), NF-κB, and fibroblast growth factors (FGFs), which coordinate communication between the epithelial and mesenchymal cells in the hair follicle (68). Although it is well-known that androgen 5α-DHT modulates the Wnt/β-catenin signaling pathway in DPCs and inhibits the transcription of hair growth genes in AGA, less is known about the behavior of other hair growth signaling pathways in AGA (1). In this study, we identified several DEGs involved in Wnt/β-catenin, NF-κB, TGF-β, BMP, and Vitamin D metabolism signaling pathways more than the original analysis by Michel et al. (5) (Supplementary I-3). Our network analysis also identified core genes that could further elucidate the pathogenesis of AGA, with a focus on the upregulated inflammatory response.

**FIGURE 7:**
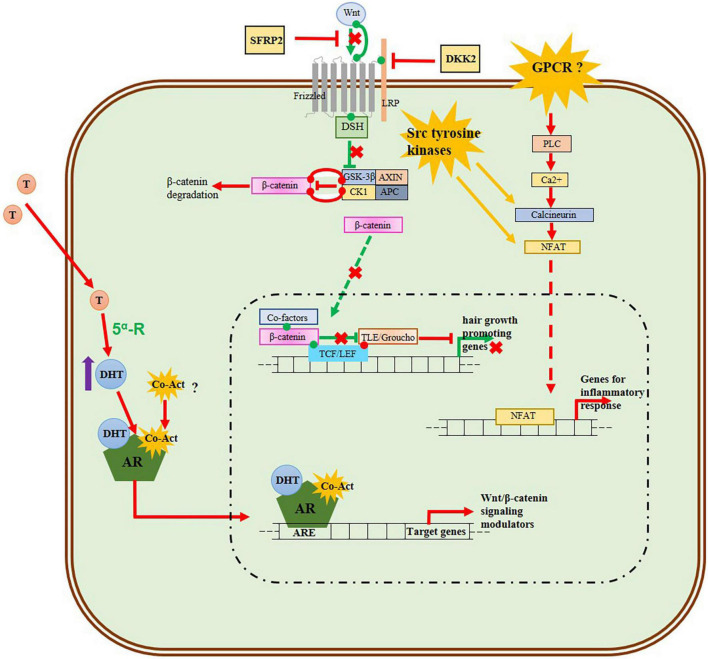
Schematic model of 5α-DHT mediated AGA in DPCs including Wnt/β-catenin signaling pathway and up-regulated inflammatory process. The green lines and arrows represent the normal activated Wnt/β-catenin signaling in the DPCs of normal-haired scalp, while the red lines and arrows denote the behavior of signaling pathways in the DPCs of balding scalp. The androgen 5α-DHT-AR complex inhibits the Wnt signaling by transcribing Wnt/β-catenin inhibitors. In our analysis genes for frizzled receptor, Wnt ligands (Wnt2b, Wnt3, Wnt5a, Wnt10b, and Wnt11), beta-catenin, LEF, and TCF are down-regulated, while the Wnt inhibitors DKK2 and SFRP2 are upregulated implying the downregulation of the normal Wnt/β-signaling pathway in AGA. The genes for phospholipase, calcineurin, and NFAT which function downstream of the non-canonical Wnt/Calcium pathway are upregulated, while the Wnt ligand for this pathway Wnt5a and frizzled receptor in the upstream are down-regulated. We propose that Src tyrosine kinase, known to interact with phospholipase and calcineurin, may activate this Wnt/Calcium pathway pathway and this needs further investigation. In addition, other Wnt ligands such as Wnt3a, Wnt4, and Wnt16 are up-regulated in our analysis and these ligands or some GPCR receptors may play a role in the activation of Wnt/calcium signaling pathway and mediate the up-regulation of NFAT which transcribes inflammatory process genes.

Conclusively, to gain a better understanding of the pathogenesis of AGA a schematic model of 5α-DHT mediated AGA in DPCs including the Wnt/β-catenin signaling pathway and the up-regulated inflammatory process is proposed in Figure 7. The nuclear factor associated with T cells (NFAT) family of transcription factors controls the expression of proinflammatory genes. The Calcineurin-NFAT signaling pathway regulates the immune system and inflammatory response (69–71). The NFAT and Wnt pathways are shown to reciprocally regulate each other constituting a non-canonical Wnt/Ca2 + /NFAT pathway in certain cells and tissues for coordinating their effects on cell growth and differentiation (71, 72). In addition, Src tyrosine kinase gene LCK are shown to interact with calcineurin and NFAT promoting NFAT activity (70, 73–75). Also the Src tyrosine kinase genes such as LCK and LYN promotes cytosolic accumulation of Ca2+ which activates calcineurin (76). In the non-canonical Wnt/Ca2+ signaling pathway calcineurin and NFAT acts downstream, but our analysis shown that the genes coding them are upregulated and the genes in the up-stream of the pathway are down-regulated. Given that the Src tyrosine kinases cross-talk with Wnt signaling and that increased activity of Src is seen during aberrant Wnt signaling in many diseases (77), we suggest that Src-tyrosine kinases may cross-talk with the androgen 5α-DHT modulated Wnt Signaling pathway and promote inflammatory response. Therefore, further investigation into the potential interactions between Src tyrosine kinase family genes, AR-5α-DHT, Wnt/β-catenin signaling, and the inflammatory response is needed to gain a more comprehensive understanding of AGA pathogenesis.

## Conclusion

Differential gene expression analysis is a powerful technique for identifying genes associated with specific conditions such as AGA. In this study, we analyzed the gene expression data from the scalps of individuals with premature AGA and normal volunteers to identify core genes associated with AGA. We identified 2,439 DEGs using a stringent criterion of log_2_FC ≥ ± 0.3 with a strict FDR value and performed gene family enrichment, GO enrichment, pathway enrichment, and motif analysis for the DEGs. Our findings indicate that down-regulated genes in AGA play significant roles in the structural makeup of the skin epidermis, hair follicle development, and hair cycle, while up-regulated genes are implicated in the innate and adaptive immune systems, cytokine signaling, and interferon signaling pathways. Moreover, we identified potential candidate genes that may contribute to AGA pathology and require further investigation. Our study also highlights the critical role of Src family tyrosine kinases in AGA pathology. Overall, this study enhances our understanding of the underlying molecular mechanisms of AGA and may lead to the development of new therapeutic strategies for treating this condition.

## Data availability statement

The datasets presented in this study can be found in online repositories. The names of the repository/repositories and accession number(s) can be found in the article/Supplementary material.

## Author contributions

AP: conceptualization, design of study, analysis and interpretation of data, and writting the manuscript. BR: conceiving and supervising the study and reviewing the manuscript. Both authors contributed to the article and approved the submitted version.

## Footnotes

1
https://ToppGene.cchmc.org/

2
http://homer.ucsd.edu/homer/microarray/index.html

3
https://string-db.org/

4
https://reactome.org/
