# Supplementary material for: Bioinformatic analysis of gene expression data reveals Src family protein tyrosine kinases as key players in androgenetic alopecia
Source: Front Med (Lausanne). 2023 Jun 9;10:1108358. doi: 10.3389/fmed.2023.1108358 (PMC10288522; doi:10.3389/fmed.2023.1108358)

Supplementary I

Bioinformatic analysis of gene expression data reveals Src family protein tyrosine kinases as key players in Androgenetic alopecia

Premanand Adaikalasamy and Reena Rajkumari Baskaran*,
Department of Integrative Biology, School of Bio Sciences and Technology,
Vellore Institute of Technology, Vellore -632014, Tamil Nadu, India.

*Corresponding author email id: b.reenarajkumari@vit.ac.in

1. Microarray Data pre-processing of 14 alopecia and 14 control samples in R using Bioconductor Packages

1a) Histogram of Raw data

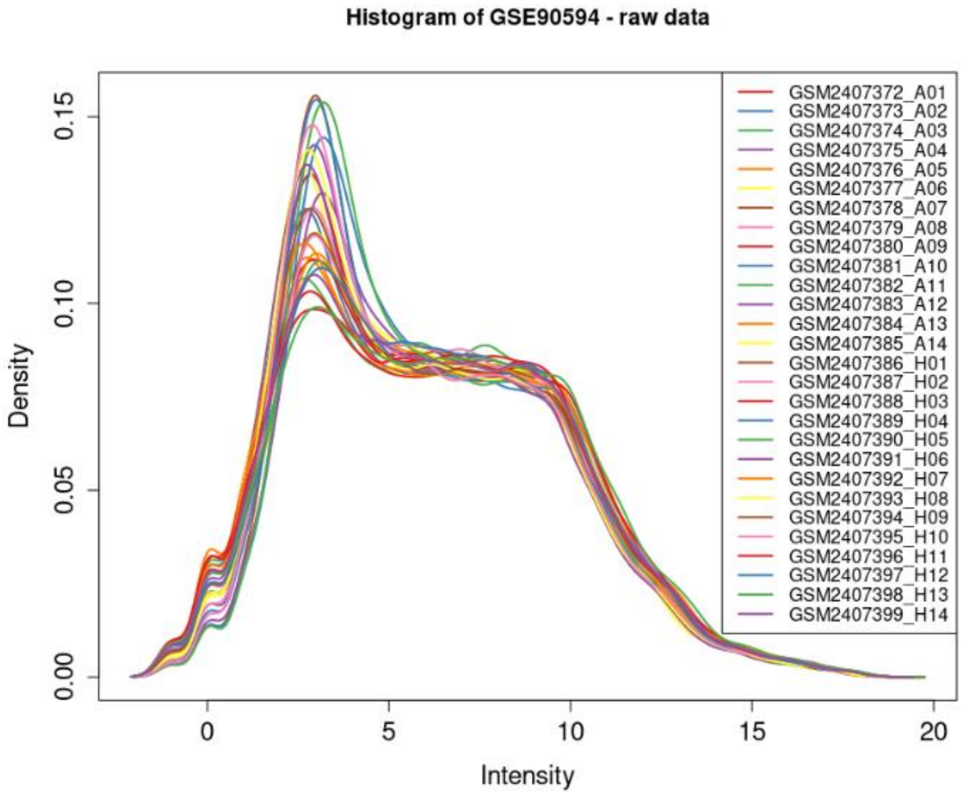

1b) Histogram of background corrected arrays (Background correction using normexp method in *limma*)

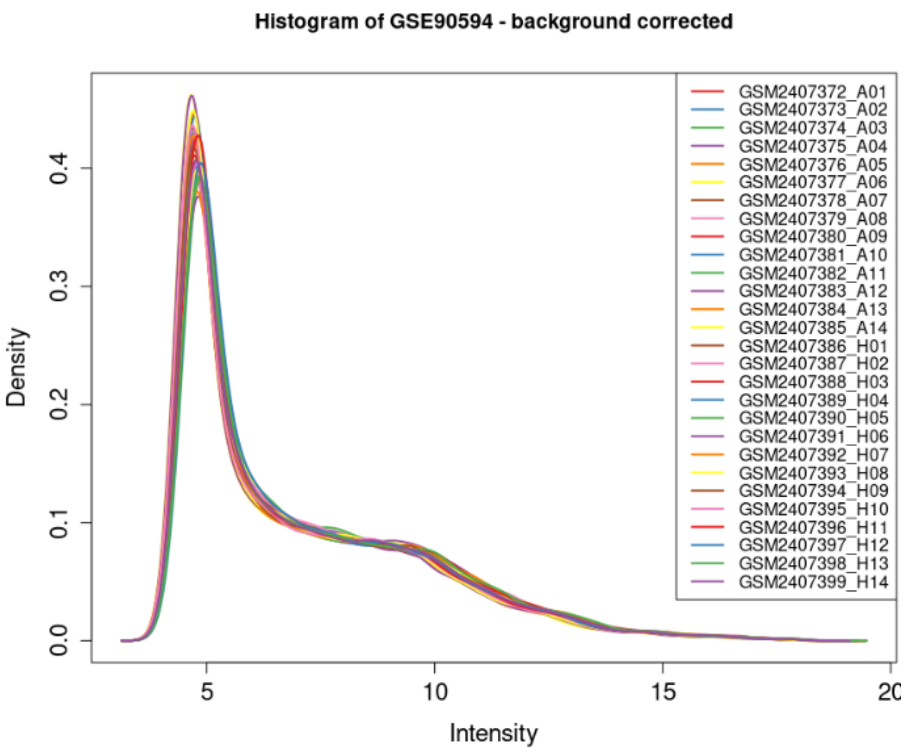

1c) Histogram of normalized arrays (normalization by quantile method in *limma*)

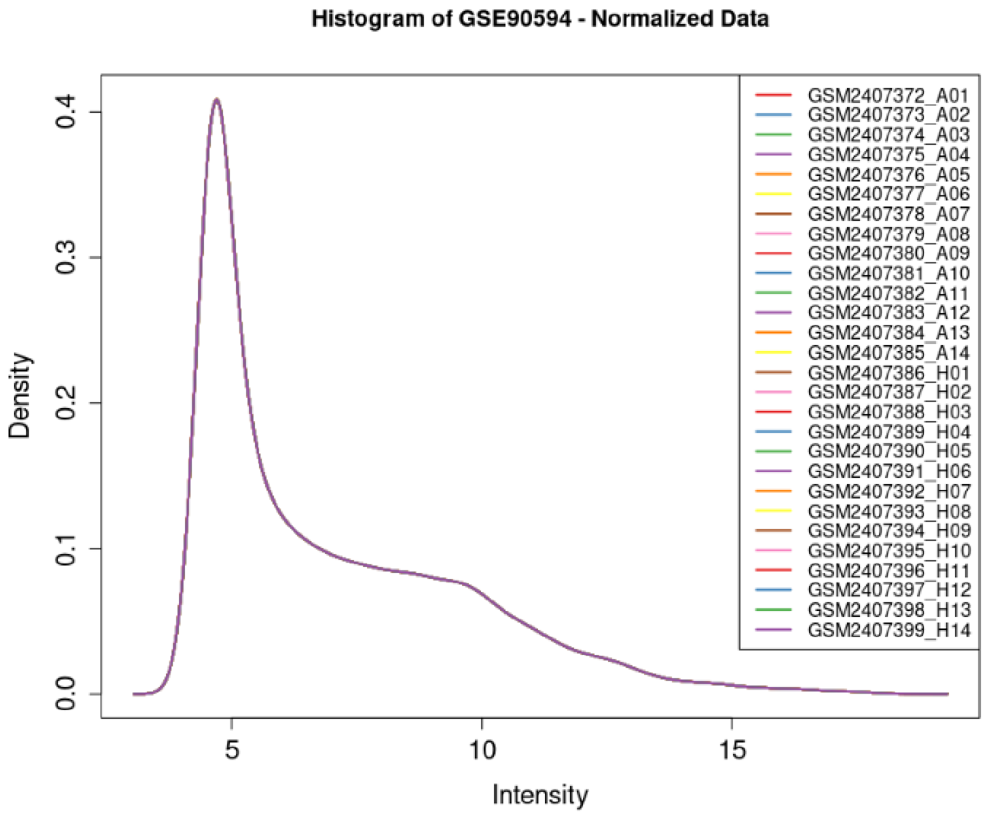

1d) Boxplot of Raw data

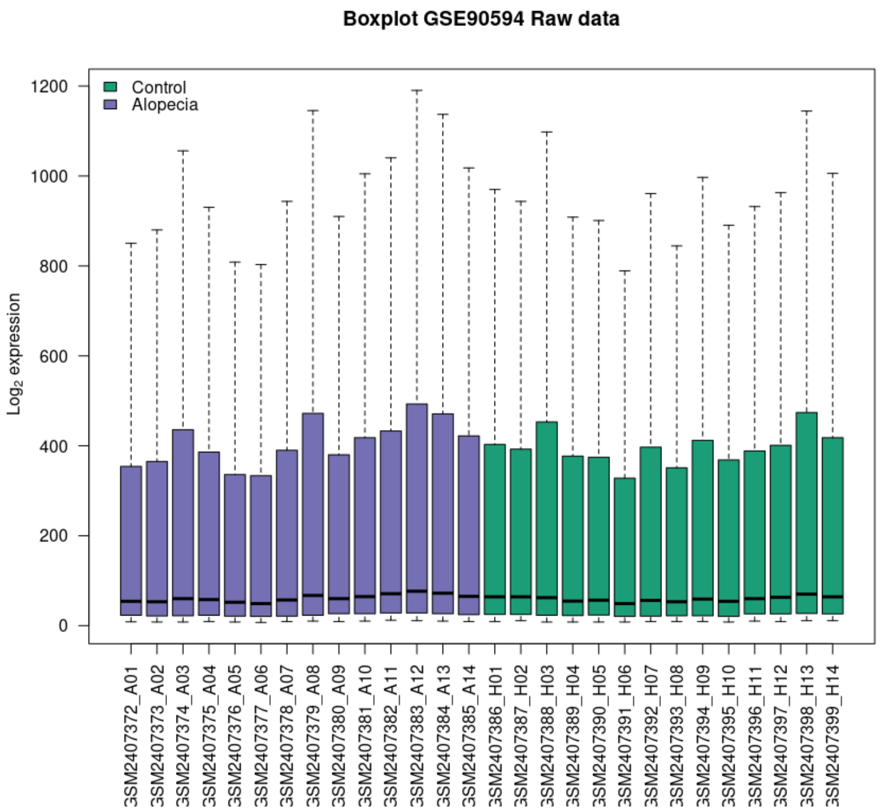

1e) Boxplot of Normalized arrays

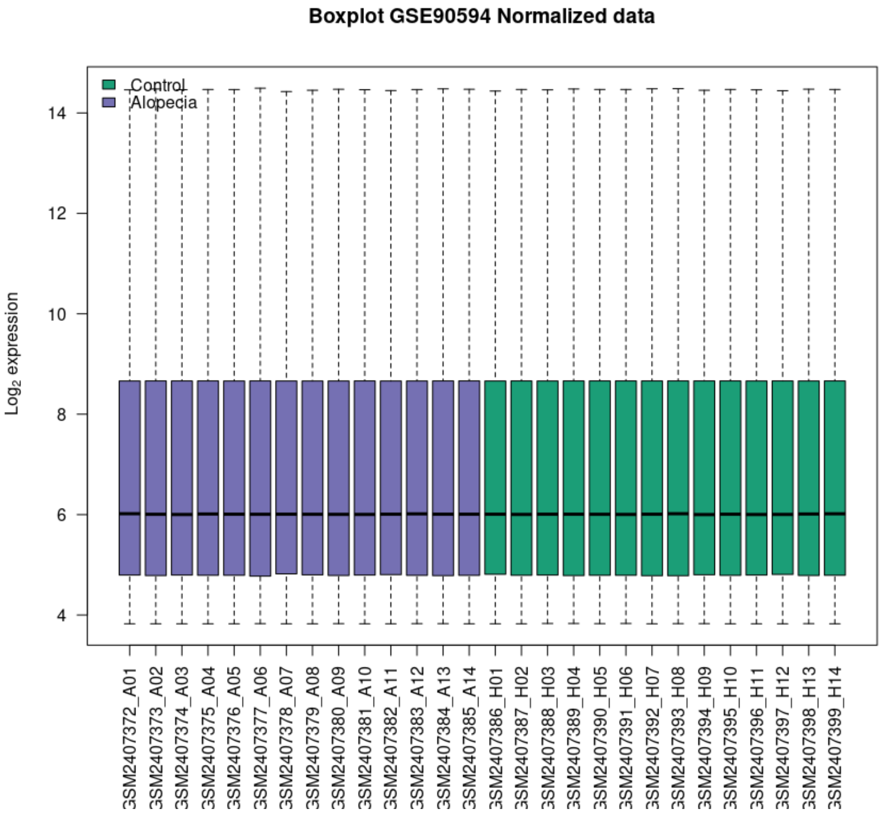

1f) Cluster dendrogram of all samples after normalized

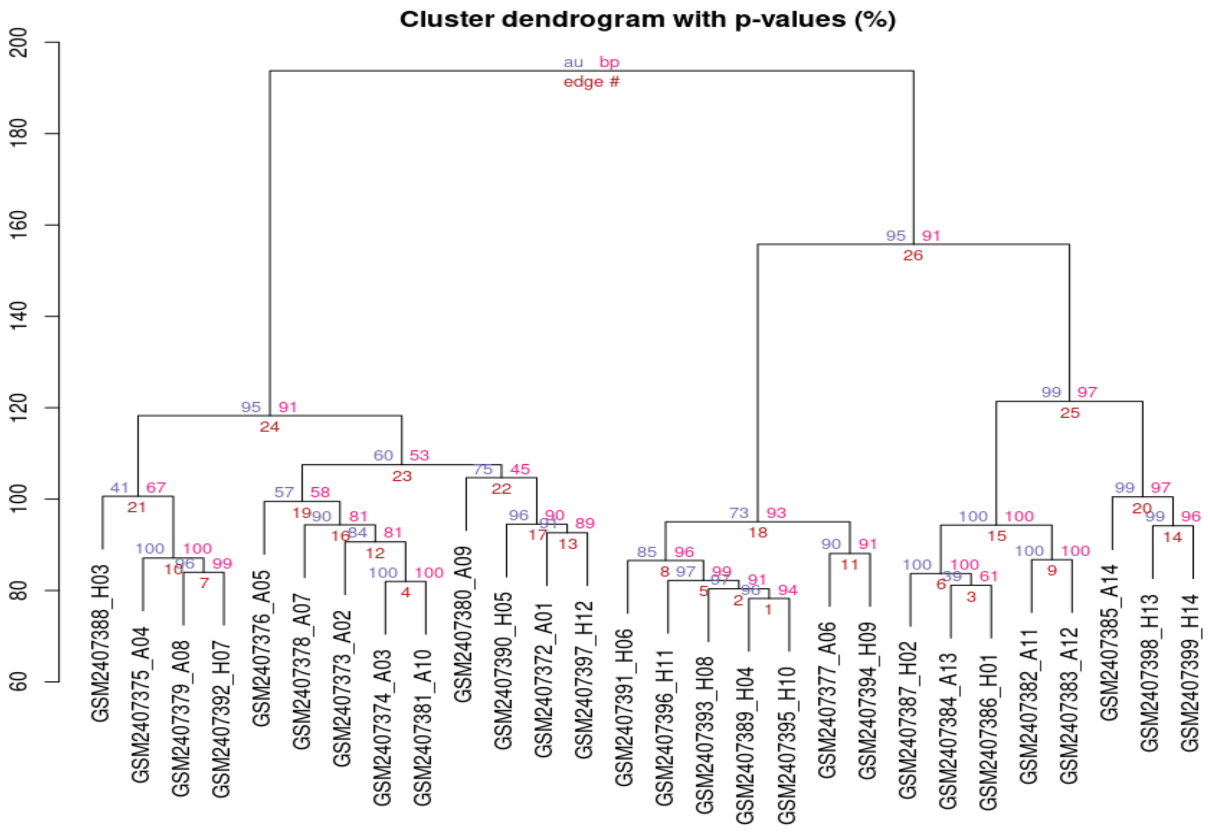

1g) UMAP plot of Raw data

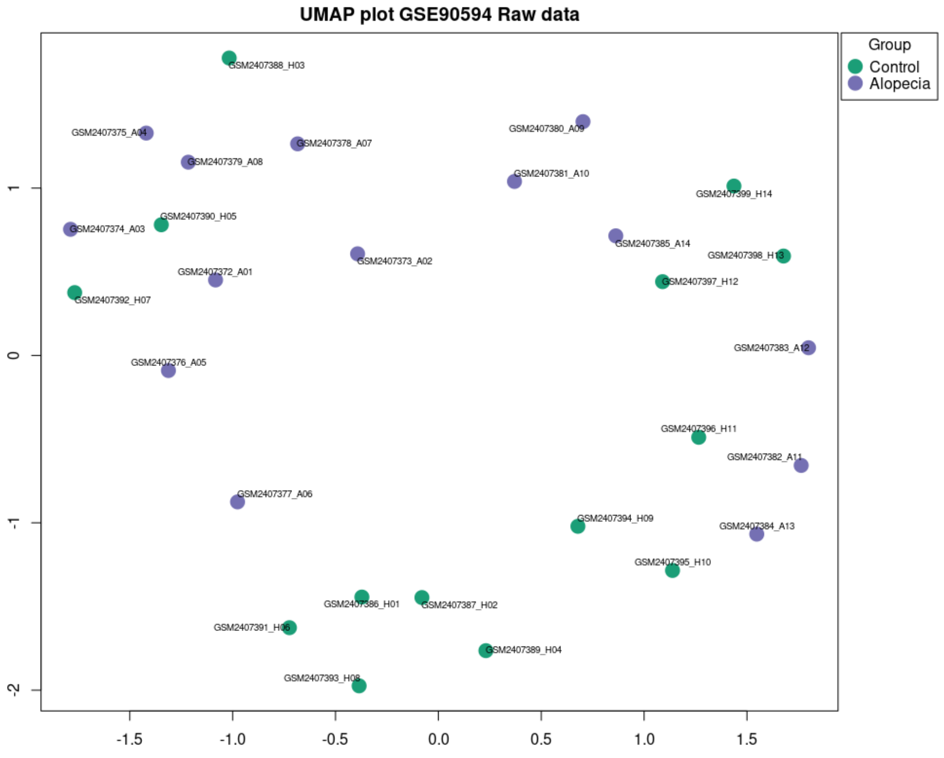

1h) UMAP plot of Normalised data

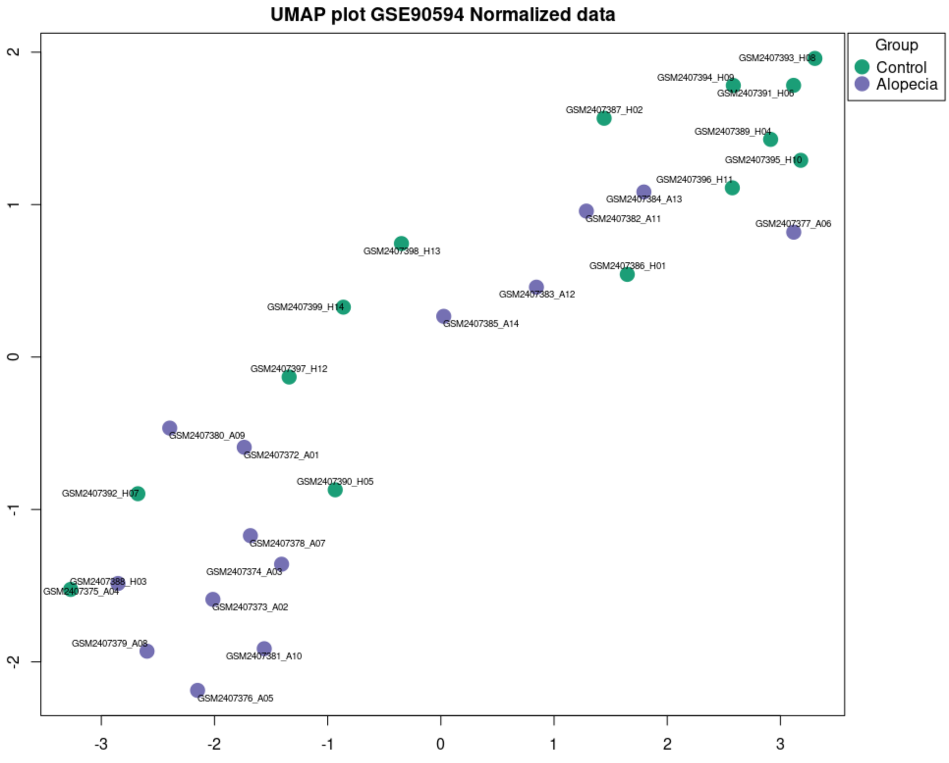

2. Microarray Data pre-processing of 9 alopecia and 10 control samples in R using Bioconductor Packages

2a) Histogram of Raw data

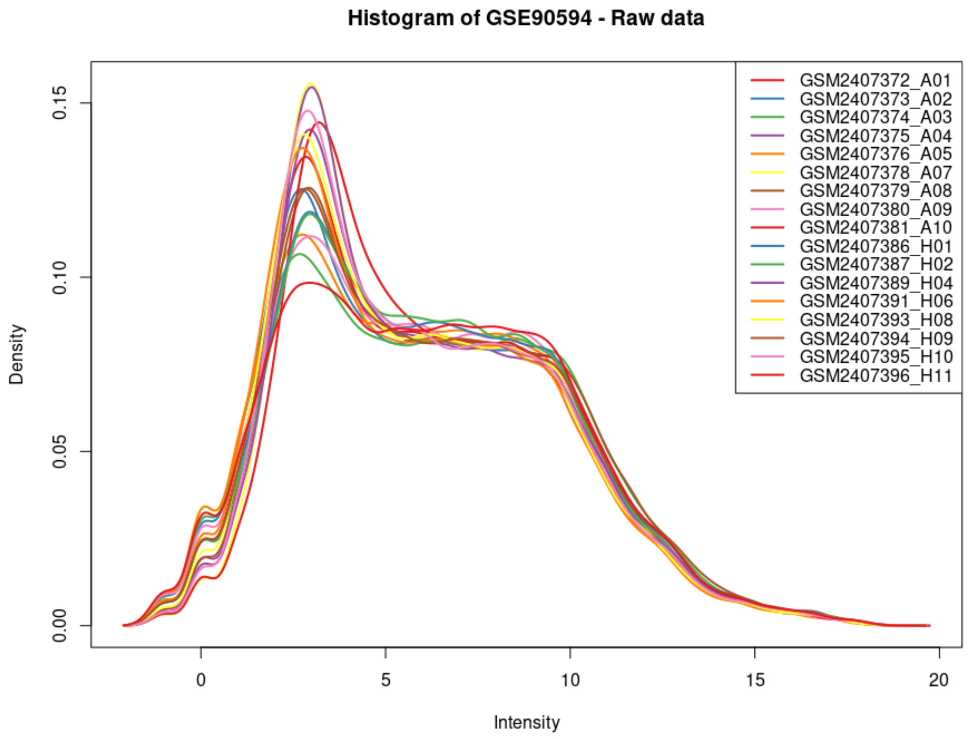

2b) Histogram of background corrected arrays (Background correction using normexp method in *limma*)

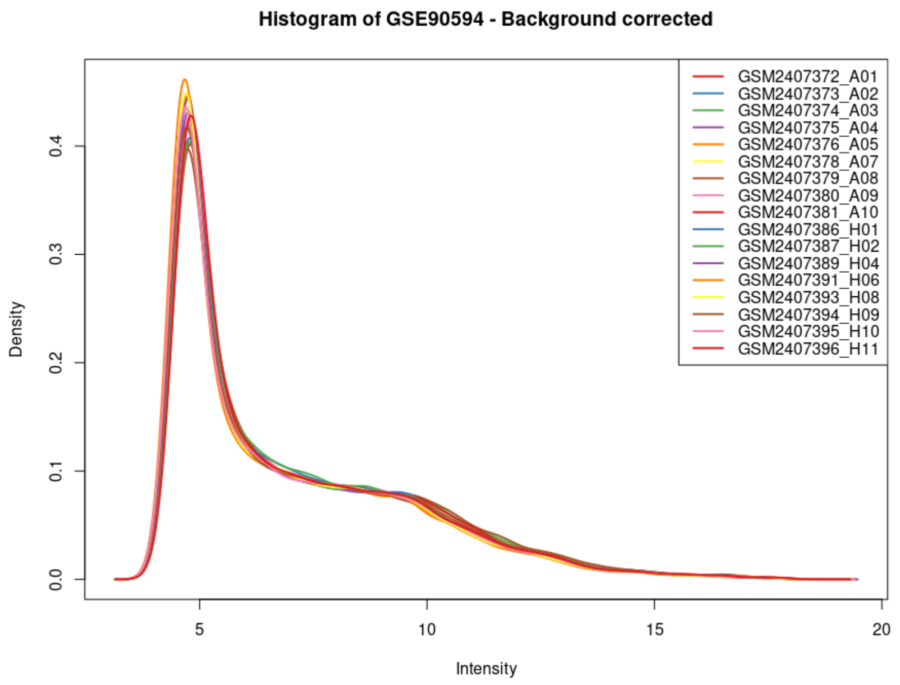

2c) Histogram of normalized arrays (normalization by quantile method in *limma*)

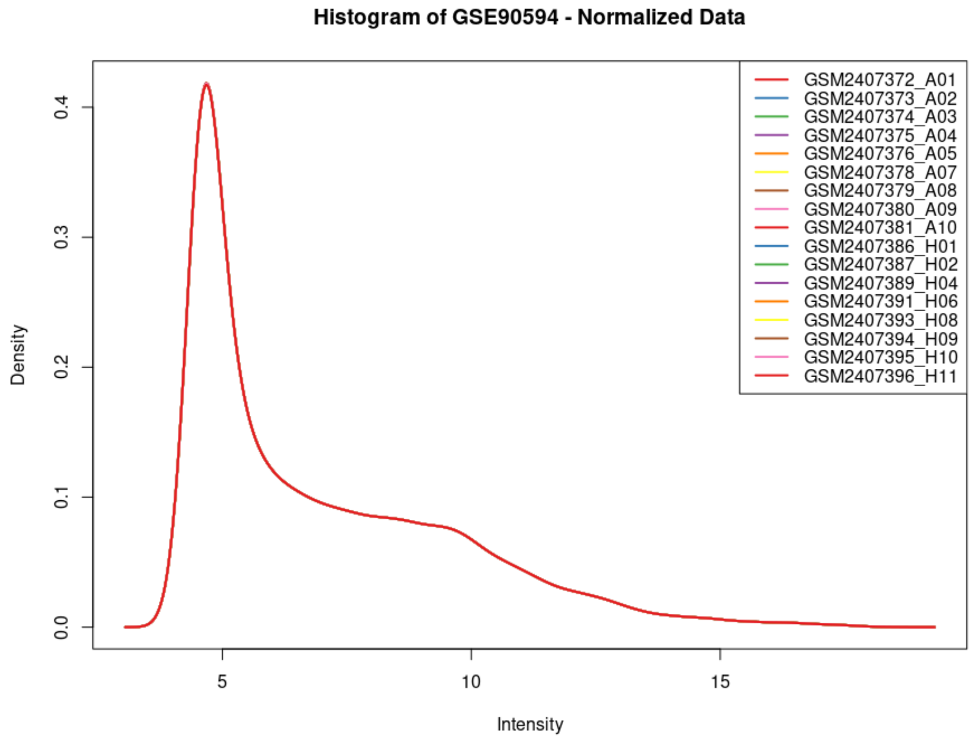

2d) Boxplot of Raw data

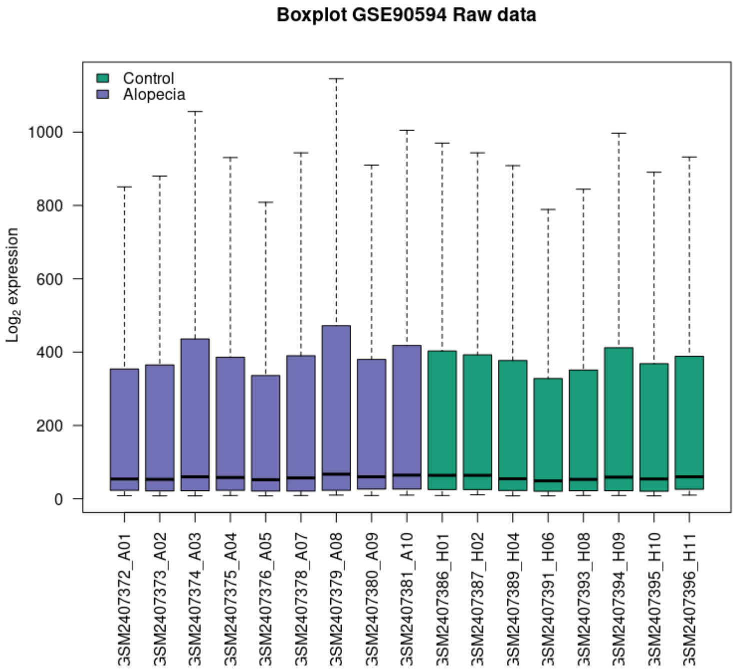

2e) Boxplot of Normalized arrays

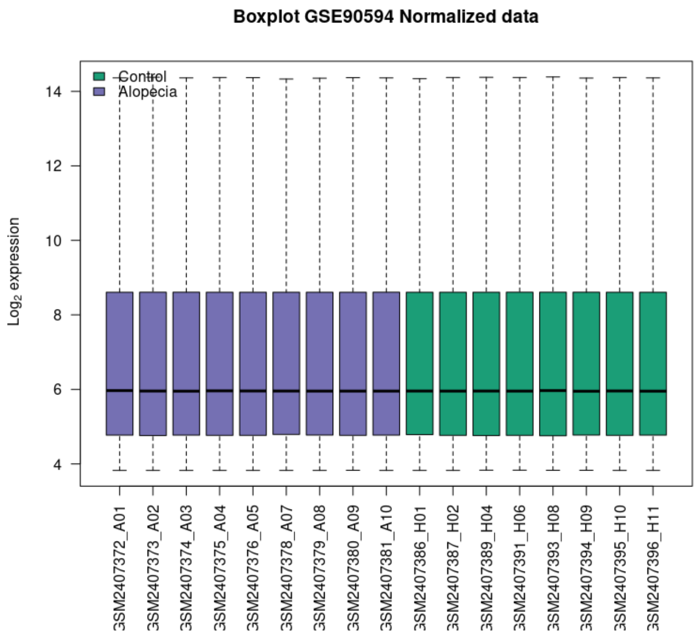

2f) Cluster dendrogram of all samples after normalized

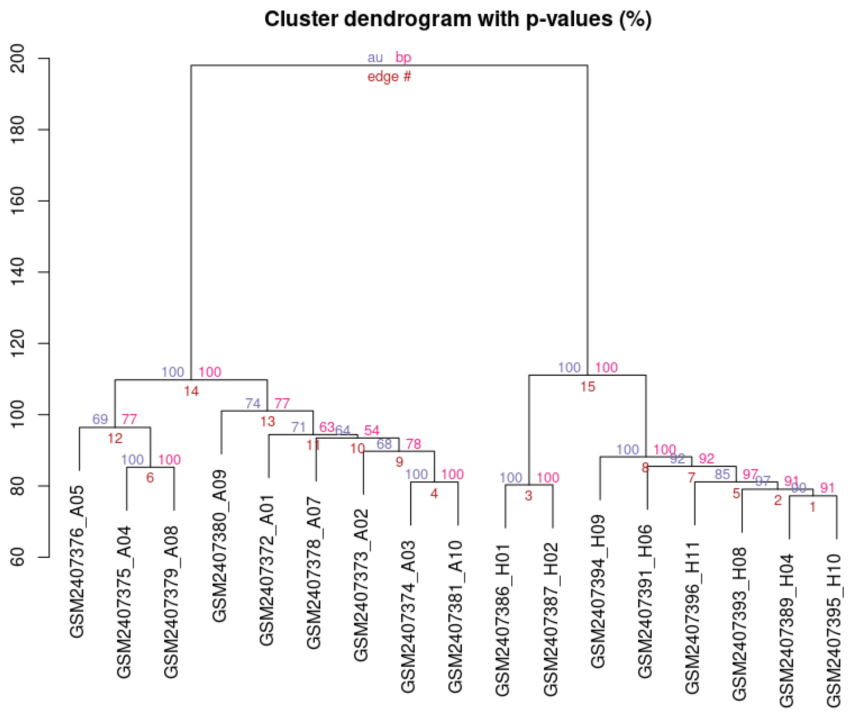

2g) UMAP plot of Raw data

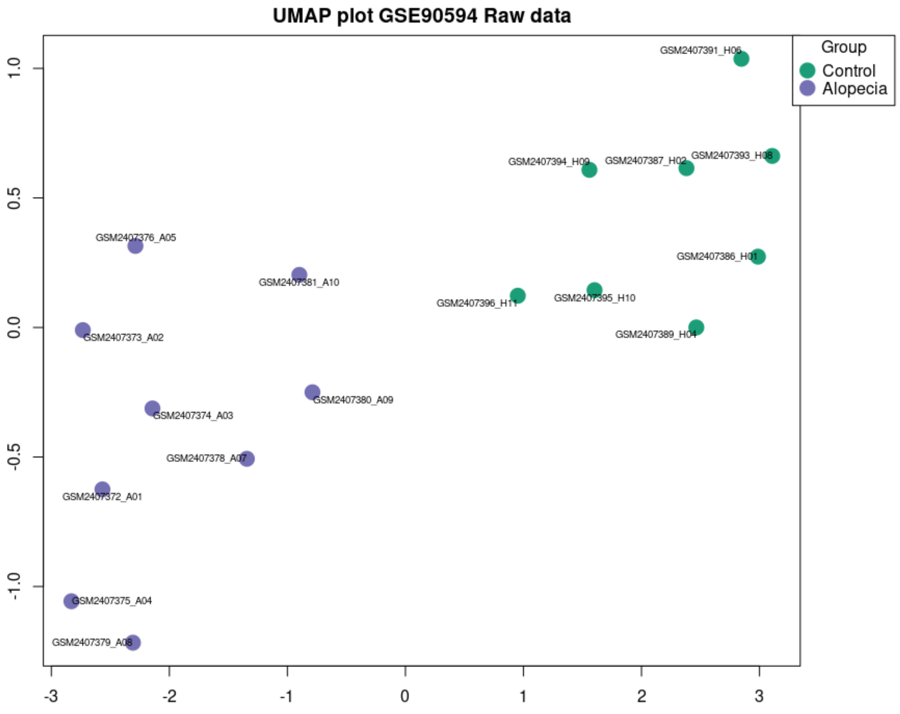

2h) UMAP plot of Normalised data

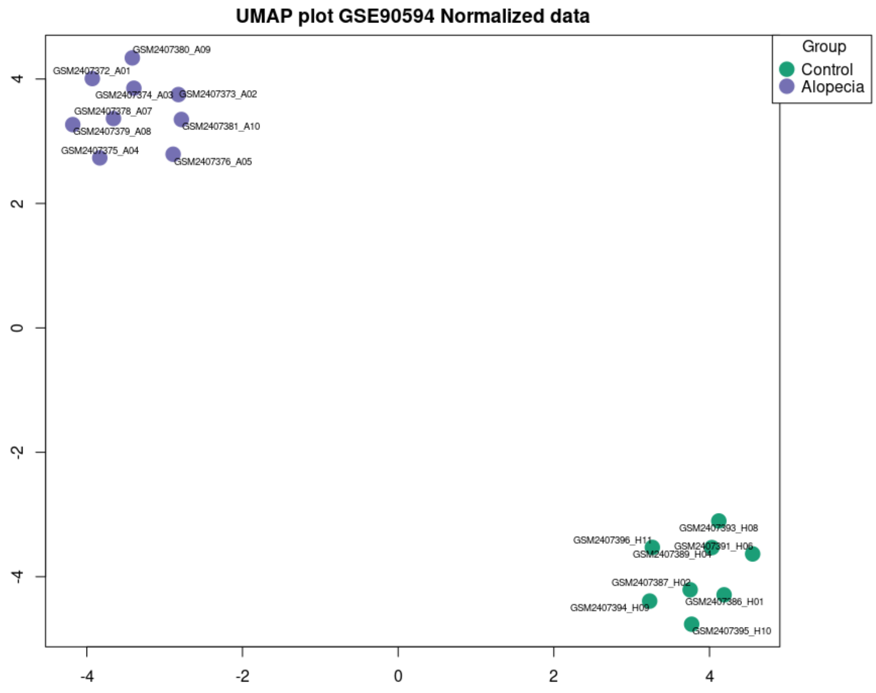

2j) Volcano plot depiciting the differential gene expression with logFC > |1| and adj.p.value < 0.05

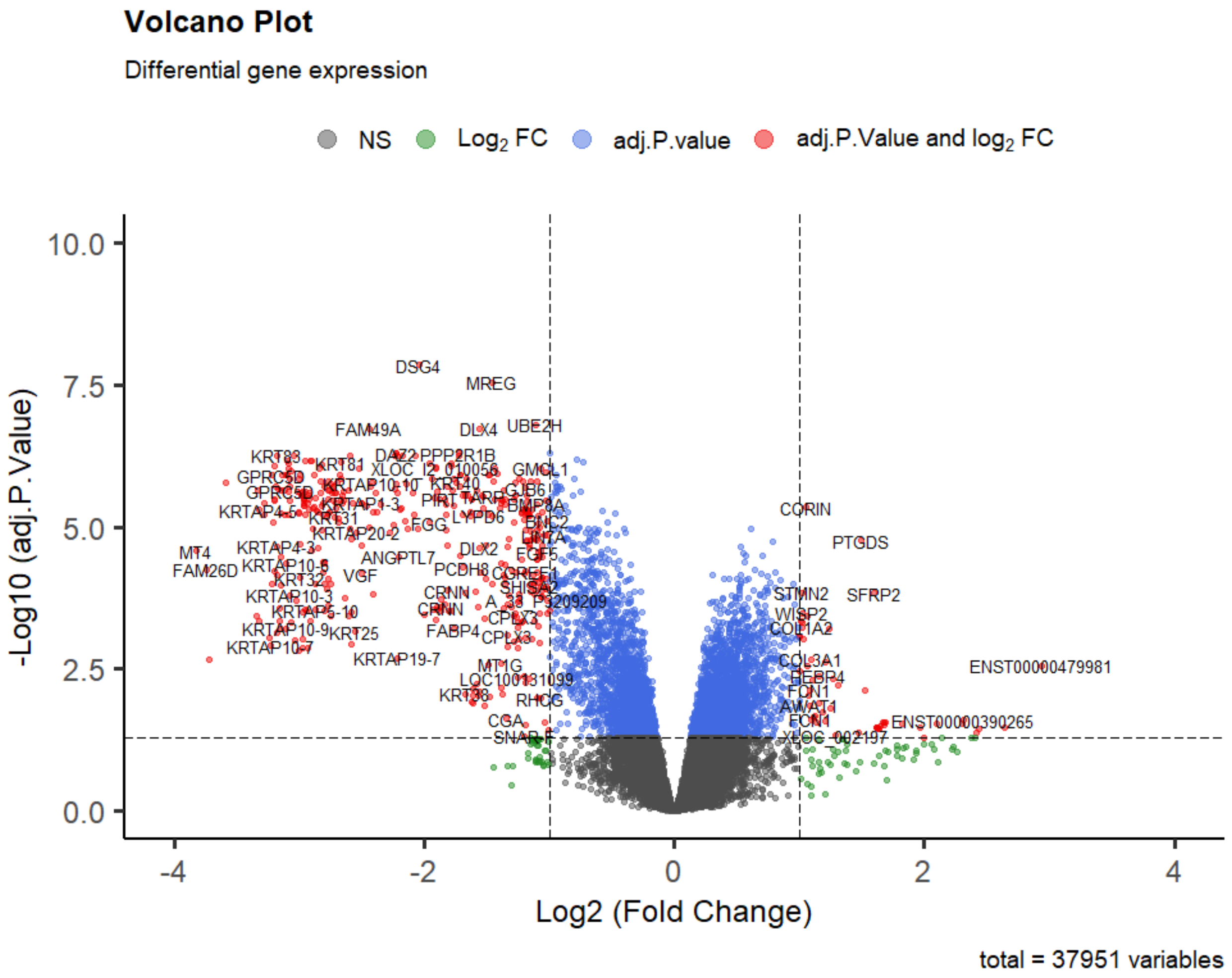

3. List of DEGs known to involved in Wnt, NF-κB, TGF-β, BMP, and Vitamin D metabolism signaling pathways.

Wnt signaling pathway related genes

Genes	log2FC
SFRP2	1.60
COL1A1	1.24
CPZ	1.11
PDE6A	0.94
NFATC4	0.62
ZBED3	0.58
CTHRC1	0.58
TLR2	0.57
ESR1	0.56
WNT3A	0.56
PLCB2	0.56
CAMK2B	0.54
PLPP3	0.53
PPARG	0.53
DAAM2	0.51
SERPINF1	0.48
ADGRA2	0.48
DACT3	0.48
WNT4	0.48
PTPRO	0.47
PSMB10	0.45
SOX7	0.42
LGR6	0.41
IGFBP4	0.40
RAC2	0.39
LRP1	0.39
WNT16	0.39
RUNX3	0.38
TBX18	0.38
H3-3B	0.37
PRKD1	0.37
ITPR1	0.36
DISC1	0.35
RACK1	0.35
PSMB9	0.34
DKK2	0.34
WNK2	0.34
CTNNBIP1	0.33
LATS2	0.32
PSMB8	0.30
BAMBI	-1.90
BMP2	-1.79
PPP2R1B	-1.73
LYPD6	-1.56
GNG4	-1.52
LEF1	-1.33
LGR5	-1.20
SHISA2	-1.16
DRD2	-0.93
CITED1	-0.85
CTNND2	-0.83
TNN	-0.77
TCF7	-0.76
MARK2	-0.73
SULF2	-0.68
WNT3	-0.68
CTNNB1	-0.65
WNT11	-0.64
TRABD2B	-0.61
FZD3	-0.61
H3C12	-0.61
GJA1	-0.59
EGR1	-0.57
APCDD1L	-0.56
FZD10	-0.55
NRARP	-0.55
SHISA3	-0.52
CELSR2	-0.52
MCC	-0.50
H4C1	-0.49
ZNF703	-0.48
H4C13	-0.48
FZD8	-0.48
MDFI	-0.47
H4C2	-0.46
PRICKLE1	-0.46
H2BC7	-0.46
WNT5A	-0.45
IGFBP2	-0.45
SDC1	-0.44
EGF	-0.44
TNIK	-0.44
SOSTDC1	-0.44
GPC6	-0.43
MITF	-0.42
WNT2B	-0.42
FZD7	-0.41
FZD6	-0.40
LGR4	-0.39
H4C4	-0.39
CLTB	-0.38
TIAM1	-0.38
WNT10B	-0.37
RACGAP1	-0.34
WLS	-0.34
CTNND1	-0.34
CELSR1	-0.34
TLE1	-0.33
H2AX	-0.32
SKP1	-0.31
GATA3	-0.30
PSMC1	-0.30

NF-κB pathway related genes

Genes	log2FC
CXCL10	0.66
IL15	0.65
ICAM1	0.55
IL32	0.54
VIM	0.51
TNFRSF1B	0.49
ERCC1	0.48
TNF	0.41
IL1R1	0.41
TNFAIP2	0.41
IRF1	0.39
ARHGDIB	0.36
STAT5A	0.35
TAP1	0.32
BMP2	-1.79
CYP27B1	-1.18
RIMS2	-1.16
THBS1	-1.10
ITGB6	-0.83
MTSS1	-0.66
HOPX	-0.64
TNC	-0.52
ZNF185	-0.47
DSG3	-0.46
WNT5A	-0.45
TIAM1	-0.38

TGF-β pathway related genes

Genes	log2FC
COL1A2	1.06
ACSBG1	0.63
MAP4K1	0.63
NFATC4	0.62
CD1E	0.58
LOX	0.55
HS3ST2	0.55
ANK1	0.52
CD163	0.50
OSR2	0.49
WNT4	0.48
TFEB	0.47
FSTL1	0.47
ABCG1	0.45
RBM47	0.45
TGFBR3	0.42
TNF	0.41
TGFBR2	0.41
HILPDA	0.41
PML	0.40
IRF1	0.39
HSPG2	0.39
FAT4	0.39
ZEB1	0.39
WNT16	0.39
RUNX3	0.38
ZEB2	0.37
PIK3CD	0.37
MN1	0.36
RAB33A	0.36
ADAM22	0.35
CSF1R	0.35
STAT3	0.34
SPTBN1	0.33
ENG	0.33
BCL6	0.31
FST	0.31
TBC1D2B	0.30
S100A3	-2.76
ATRNL1	-2.05
BAMBI	-1.90
BMP2	-1.79
LEF1	-1.33
RIMS2	-1.16
THBS1	-1.10
CITED1	-0.85
ITGB6	-0.83
CYP24A1	-0.79
SMAD7	-0.75
LTBP2	-0.74
TMC7	-0.70
WNT3	-0.68
UBE2D1	-0.67
CDKN2B	-0.66
ID3	-0.66
CTNNB1	-0.65
WNT11	-0.64
SMAD6	-0.60
BMP4	-0.57
TNC	-0.52
RNF19B	-0.52
ENTPD7	-0.52
TMCC2	-0.50
SEC14L2	-0.48
FNBP1L	-0.45
PLXNA2	-0.45
EGF	-0.44
SKIL	-0.44
MITF	-0.42
F11R	-0.42
CDK1	-0.41
LOXL2	-0.41
FGF1	-0.40
LGR4	-0.39
PDLIM4	-0.37
ZFP36L1	-0.37
CCNB2	-0.34
ADAMTS1	-0.33
COL16A1	-0.33
SLC5A3	-0.31
SPSB1	-0.31
SKP1	-0.31
GATA3	-0.30
BAG4	-0.30
MPPED2	-0.30

BMP pathway related genes

Genes	log2FC
SFRP2	1.60
CHRD	0.60
PPARG	0.53
WIPF1	0.51
FSTL1	0.47
RPGRIP1	0.46
KIF17	0.43
TGFBR3	0.42
ENG	0.33
RTN4	0.31
FST	0.31
ANGPTL7	-2.21
COMP	-2.19
DSG4	-2.04
BAMBI	-1.90
BMP2	-1.79
LEF1	-1.33
BMP8A	-1.10
DLX1	-0.99
HES5	-0.88
PCSK6	-0.87
RGMA	-0.75
SMAD7	-0.75
ADAMTS7	-0.70
MT1B	-0.69
SESN3	-0.67
UBE2D1	-0.67
PPIG	-0.66
SMAD6	-0.60
BMP7	-0.59
EGR1	-0.57
BMP4	-0.57
AHSG	-0.54
TGFB2	-0.51
MSX1	-0.51
LRP2	-0.45
WNT5A	-0.45
SKIL	-0.44
SOSTDC1	-0.44
BMPER	-0.42
PDCD4	-0.40
FAM83G	-0.40
CRKL	-0.35
SORL1	-0.34
KIF1B	-0.31
GATA3	-0.30
MPPED2	-0.30

Vitamin D metabolism related genes

Genes	log2FC
ACOX2	0.62
CYP2R1	0.44
CYP27A1	0.42
TNF	0.41
DHCR7	0.40
EFL1	-1.47
CYP27B1	-1.18
CYP24A1	-0.79
VDR	-0.47
LRP2	-0.45

4. Top 3 clusters identified in the STRING protein-protein interaction (PPI) network.

4a. Cluster 1

Cluster 1 consisted of 53 nodes and 257 edges. The up-regulated DEGs are represented in green, while the down-regulated DEGs are in red. The hub node identified from the STRING PPI network is represented by a big hexagon-bordered node**.**

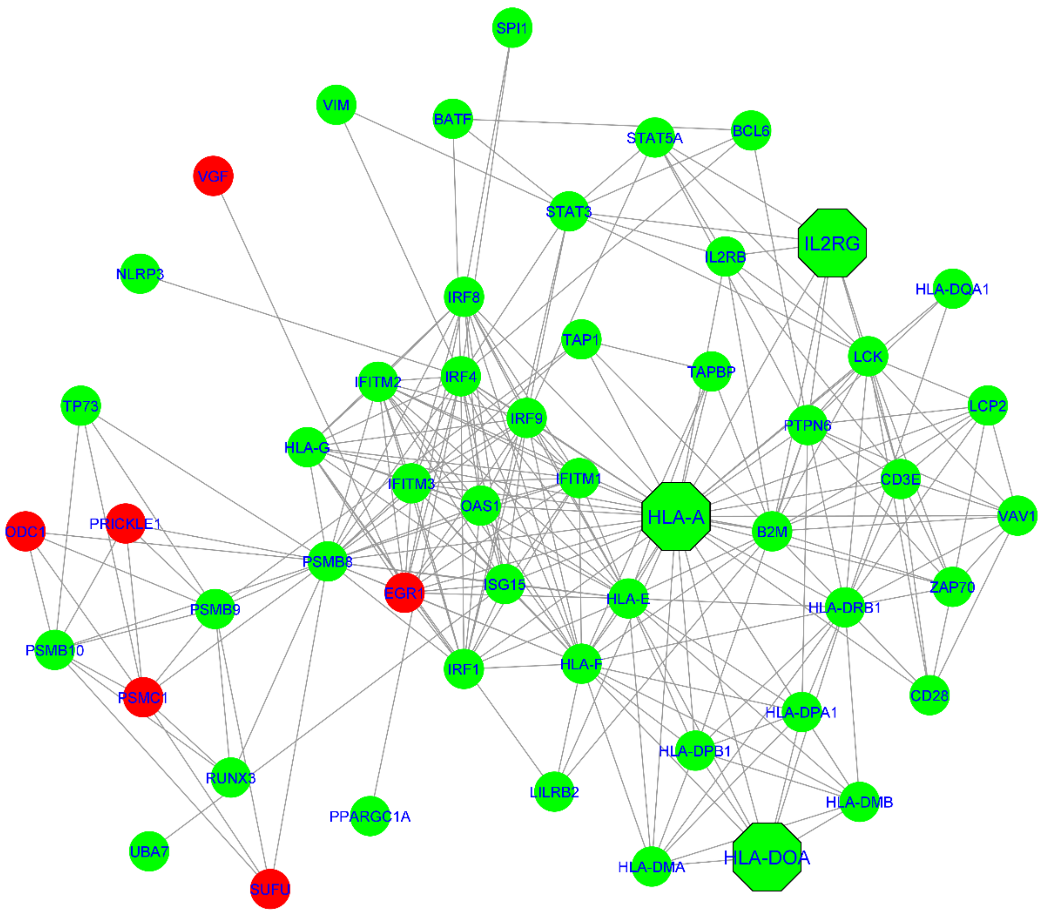

4b. Cluster 2

Cluster 2 consisted of 63 nodes and 222 edges. The up-regulated DEGs are represented in green, while the down-regulated DEGs are in red. The hub node identified from the STRING PPI network is represented by a big hexagon-bordered node**.**

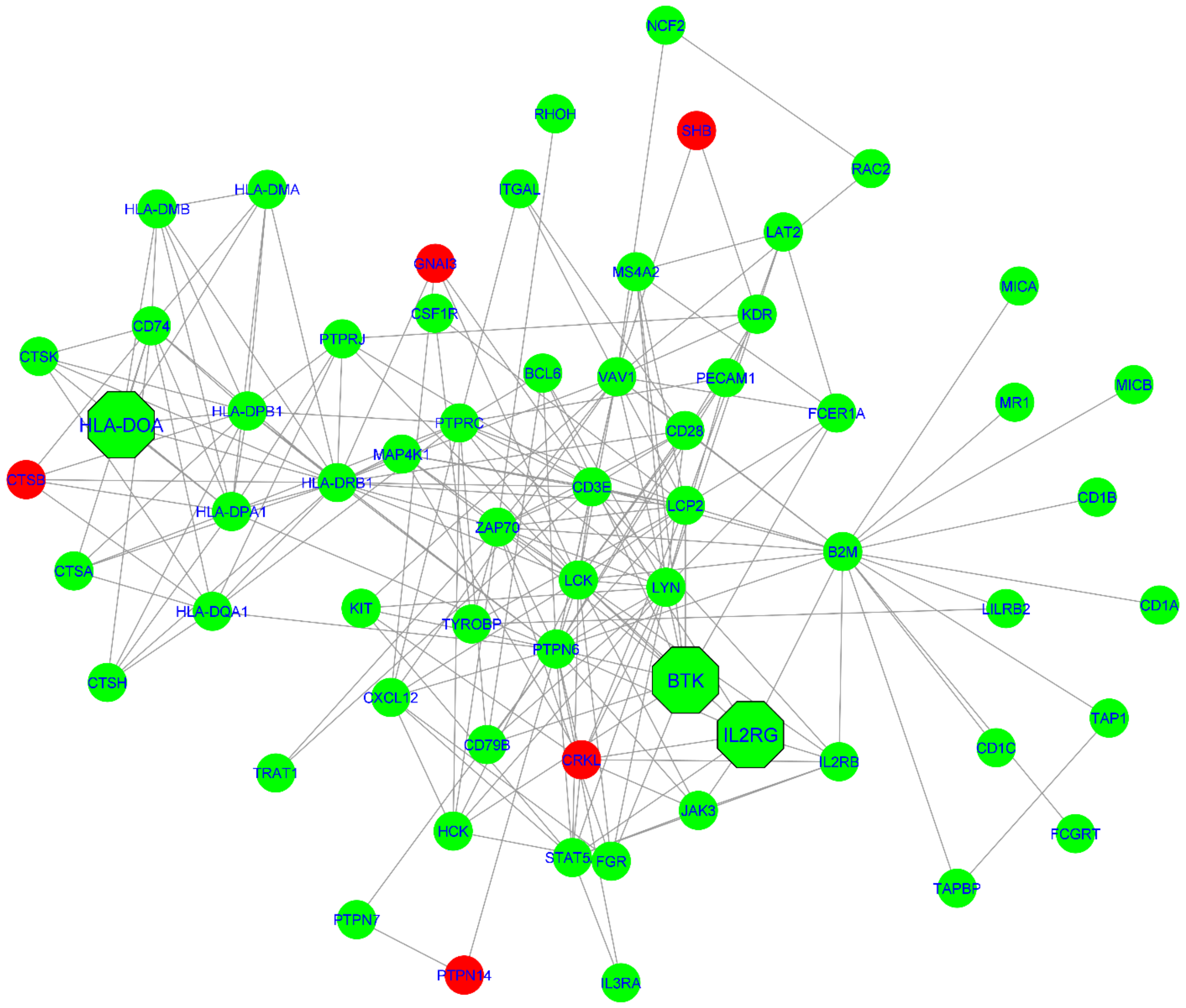

4c. Cluster 3

Cluster 3 consisted of 101 nodes and 294 edges. The up-regulated DEGs are represented in green, while the down-regulated DEGs are in red. The hub node identified from the STRING PPI network is represented by a big hexagon-bordered node**.**

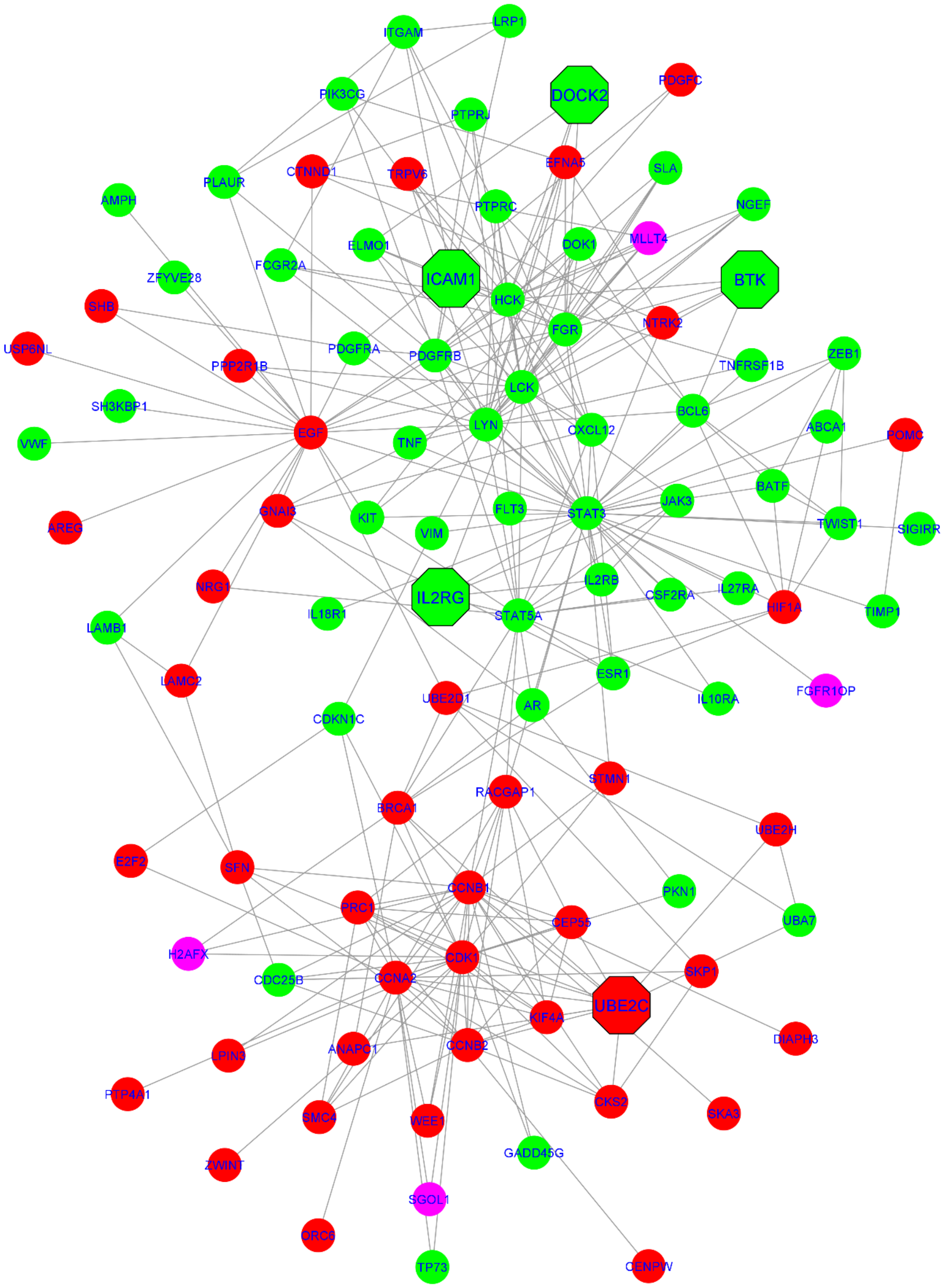

5. Top 3 clusters identified in the reactome Functional Interaction (FI) network.

5a. Cluster 1

Cluster 1 consisted of 189 nodes and 1171 edges. The up-regulated DEGs are represented in green, while the down-regulated DEGs are in red. The hub node identified from the reactome FI network is represented by a big hexagon-bordered node.

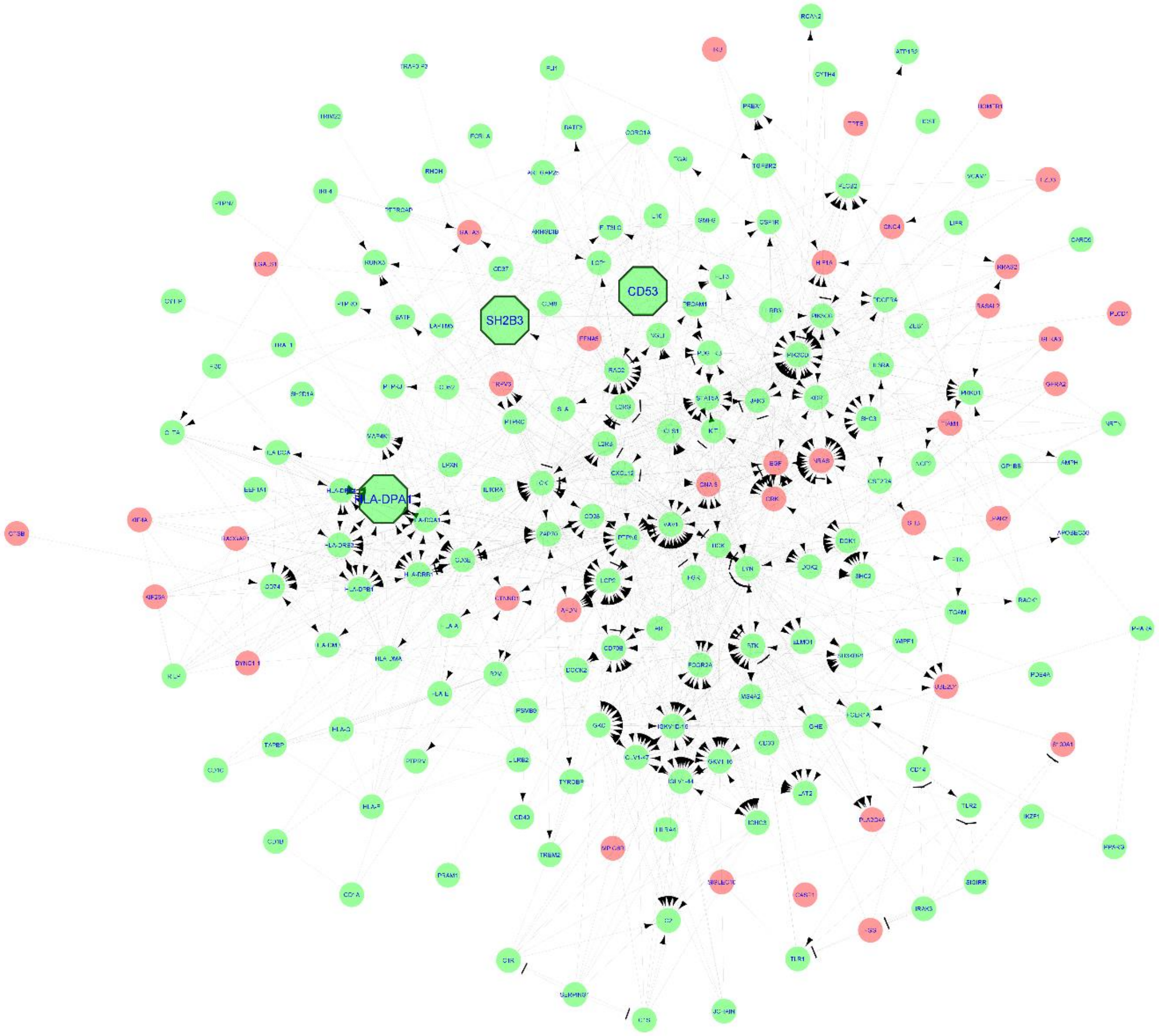

5b. Cluster 2

Cluster 2 consisted of 55 nodes and 260 edges. The up-regulated DEGs are represented in green, while the down-regulated DEGs are in red. The hub node identified from the reactome FI network is represented by a big hexagon-bordered node**.**

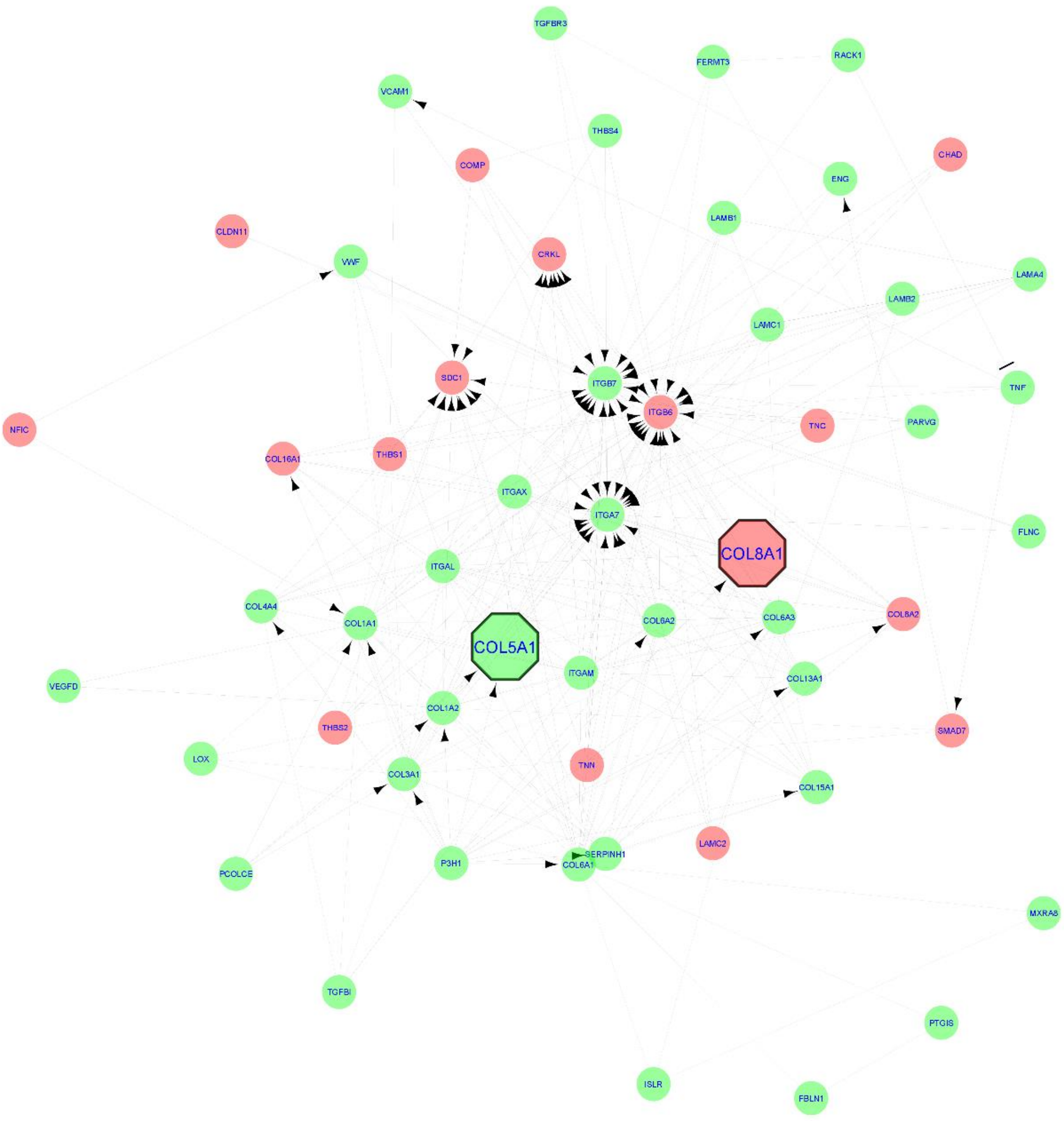

5c. Cluster 3

Cluster 3 consisted of 216 nodes and 873 edges. The up-regulated DEGs are represented in green, while the down-regulated DEGs are in red. The hub node identified from the reactome FI network is represented by a big hexagon-bordered node**.**

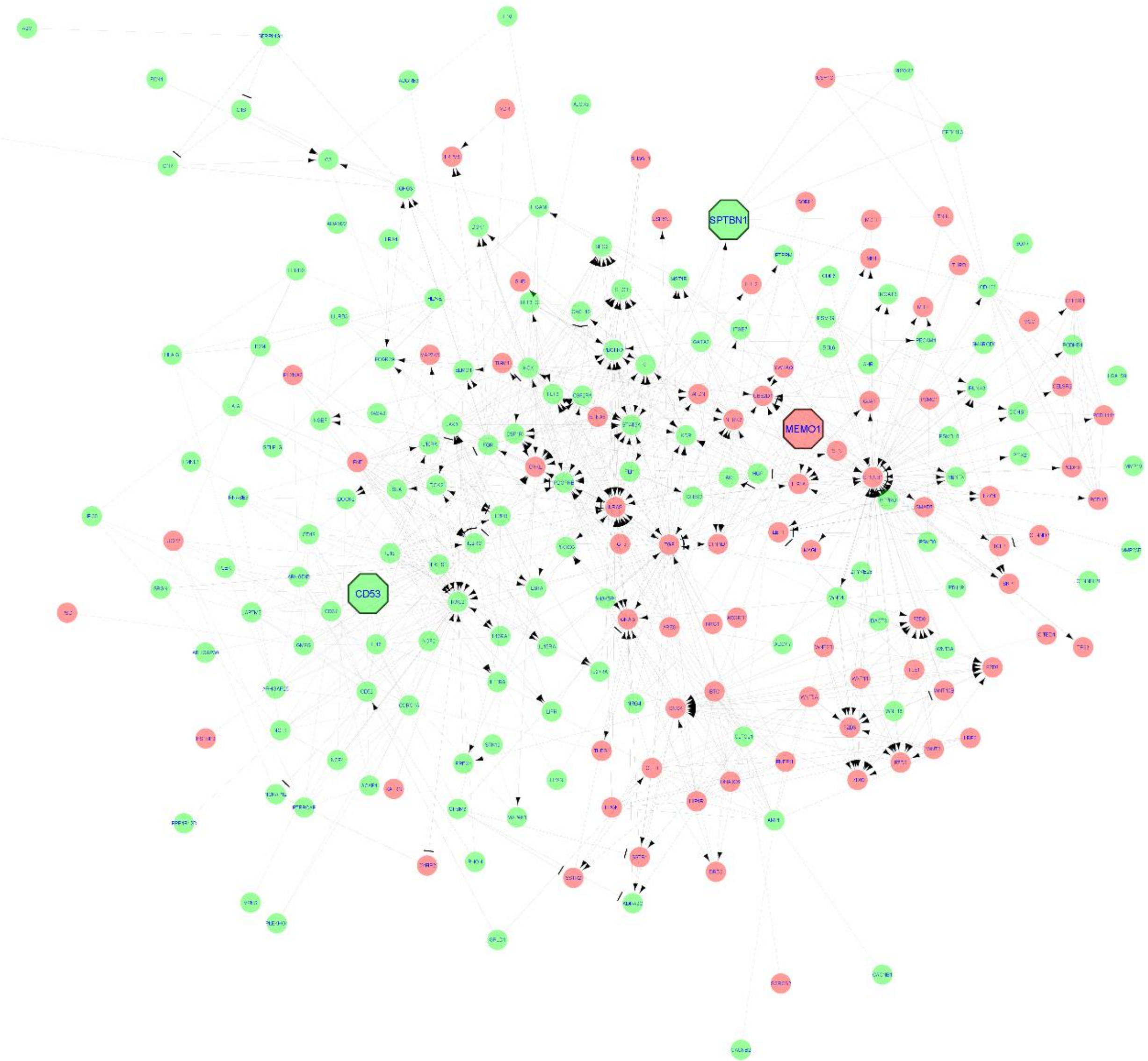

Supplement: Supplementary file 1 [file Data_Sheet_1.pdf]
